# The role of inflammatory mediators and matrix metalloproteinases (MMPs) in the progression of osteoarthritis

**DOI:** 10.1016/j.bbiosy.2024.100090

**Published:** 2024-02-21

**Authors:** Anwesha Mukherjee, Bodhisatwa Das

**Affiliations:** Department of Biomedical Engineering, Indian Institute of Technology Ropar, India

**Keywords:** Osteoarthritis, Inflammation, Oxidative damage, Matrix degradation, MMP-Inhibitor

## Abstract

Osteoarthritis (OA) is a chronic musculoskeletal disorder characterized by an imbalance between (synthesis) and catabolism (degradation) in altered homeostasis of articular cartilage mediated primarily by the innate immune system. OA degenerates the joints resulting in synovial hyperplasia, degradation of articular cartilage with damage of the structural and functional integrity of the cartilage extracellular matrix, subchondral sclerosis, osteophyte formation, and is characterized by chronic pain, stiffness, and loss of function. Inflammation triggered by factors like biomechanical stress is involved in the development of osteoarthritis. In OA apart from catabolic effects, anti-inflammatory anabolic processes also occur continually. There is also an underlying chronic inflammation present, not only in cartilage tissue but also within the synovium, which perpetuates tissue destruction of the OA joint. The consideration of inflammation in OA considers synovitis and/or other cellular and molecular events in the synovium during the progression of OA. In this review, we have presented the progression of joint degradation that results in OA. The critical role of inflammation in the pathogenesis of OA is discussed in detail along with the dysregulation within the cytokine networks composed of inflammatory and anti-inflammatory cytokines that drive catabolic pathways, inhibit matrix synthesis, and promote cellular apoptosis. OA pathogenesis, fluctuation of synovitis, and its clinical impact on disease progression are presented here along with the role of synovial macrophages in promoting inflammatory and destructive responses in OA. The role of interplay between different cytokines, structure, and function of their receptors in the inter-cellular signaling pathway is further explored. The effect of cytokines in the increased synthesis and release of matrix-decomposing proteolytic enzymes, such as matrix metalloproteinase (MMPs) and a disintegrin-like and metalloproteinase with thrombospondin motif (ADAMTS), is elaborated emphasizing the potential impact of MMPs on the chondrocytes, synovial cells, articular and periarticular tissues, and other immune system cells migrating to the site of inflammation. We also shed light on the pathogenesis of OA via oxidative damage particularly due to nitric oxide (NO) via its angiogenic response to inflammation. We concluded by presenting the current knowledge about the tissue inhibitors of metalloproteinases (TIMPs). Synthetic MMP inhibitors include zinc binding group (ZBG), non-ZBG, and mechanism-based inhibitors, all of which have the potential to be therapeutically beneficial in the treatment of osteoarthritis. Improving our understanding of the signaling pathways and molecular mechanisms that regulate the MMP gene expression, may open up new avenues for the creation of therapies that can stop the joint damage associated with OA.

## Highlights

• Osteoarthritis (OA) traditionally known to be a disease prevalent in geriatric populations and mechanical injury is currently observed to have significant connections relevant to inflammation and free radical biology.
• Pro-and anti-inflammatory cytokines and their involvement in triggering the enhanced matrix breakdown through excessive matrix metalloproteinases (MMPs) activation is a hallmark of osteoarthritic cartilage lesions.
• Imbalance between MMP/TIMP (tissue inhibitors of metalloproteinases) ratio increased free radical generation ultimately precipitate into aggravated OA condition.
• MMP inhibitors have been suggested as therapeutic treatments for osteoarthritis.

## Graphical abstract

**Figure:**
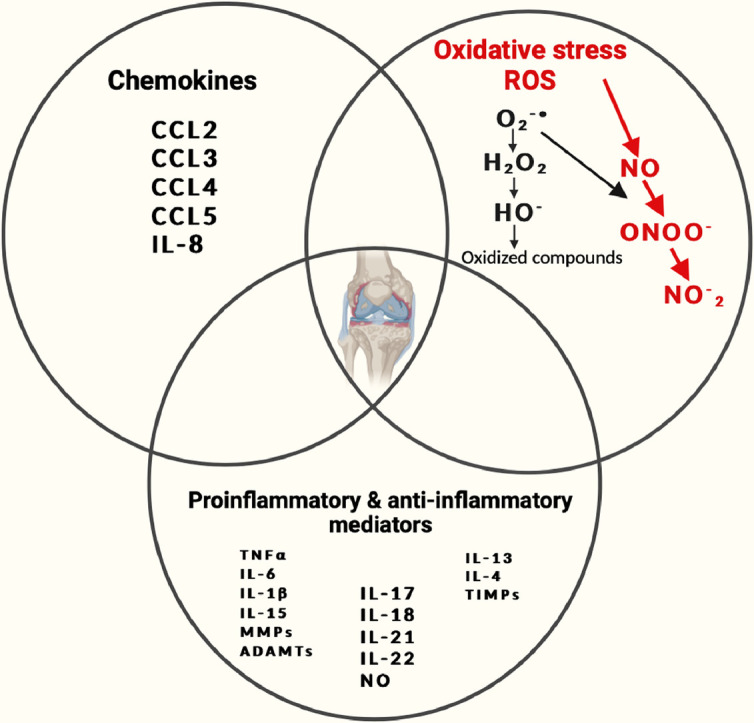

## Introduction

The articular cartilage becomes damaged due to overuse or pathological processes including abnormal mechanical loading or injury. A pathologic change occurs in the cartilage, subchondral bone, and synovium of the joints due to the chronic, progressive, degenerative joint disease, osteoarthritis (OA) with an unknown etiology [1]. It affects an aging population globally and lowers the quality of life while also making people disabled. In addition, OA causes an increased socioeconomic burden to the patients and society as a whole [2]. According to the American College of Rheumatology, osteoarthritis is defined as a “heterogeneous group of conditions that lead to joint symptoms and signs which are associated with defective integrity of articular cartilage, in addition to related changes in the underlying bone at the joint margins” [3]. The bones rub against each other in joints including the knees, hands, hips, and spine causing stiffness, pain, and reduced movement. Therefore, OA is a musculoskeletal condition defined by cartilage degradation (thinning or a decrease in articular cartilage (AC) thickness). OA also damages muscles, ligaments, and menisci. Additional pain and potentially damaging surrounding tissues are created due to subchondral bone sclerosis (bone thickening), the formation of bone spurs or osteophytes (bone outgrowth on the joint margin), and modification of the synovial fluid composition.

Clinically, joint pain, tenderness, crepitus, stiffness, and limitation of movement with occasional effusion and varying degrees of local inflammation are the hallmarks of OA. Since AC has little or no ability for regeneration, the lack of effective repair contributes to the widespread damage related to OA. The structural changes in an OA joint are thought to be due to an imbalance in degradative (catabolic) and synthetic (anabolic) activity resulting in excessive production of matrix degrading enzymes and insufficient matrix repair. Contributing factors to the imbalance in homeostasis include the direct effects of mechanical loading on joint tissues, autocrine and paracrine signaling initiated by cytokines and the damaged matrix itself (matrix fragments), and an alteration in the phenotype of the chondrocytes which are the cells in the articular cartilage responsible for maintenance of the cartilage matrix.

The adult human skeletal system consists of the 206 identified bones linked by cartilage, tendons, ligaments, and three different types of joints:diarthroses/synovial joints (which are freely movable) amphiarthroses/cartilaginous joints (which are somewhat movable), and synarthroses/fibrous joints (which are immovable). Detailed discussions about joints will be obtained in references [4], [5], [6], [7].

**Fig. 1:**
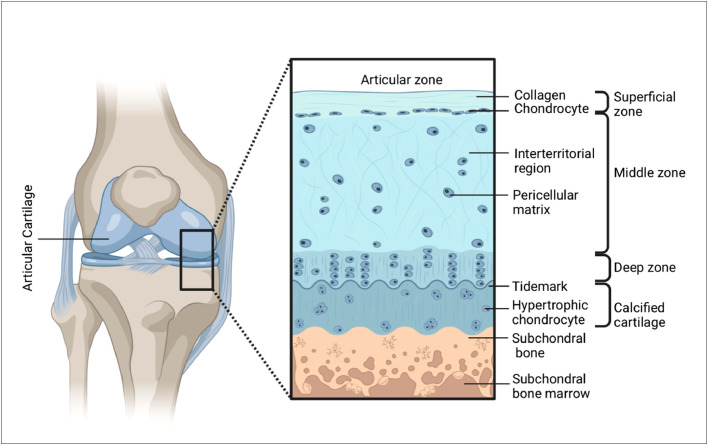
Architecture of cartilage tissue.

Articular cartilage (AC) is a highly specialized connective and weight-bearing tissue that covers the ends of articulating bones in the diarthrodial joints of the body, giving support, flexibility, and strength [8], [9], [10], [11], [12], [13], [14]. It is made up of a thick extracellular matrix (ECM) with chondrocytes dispersed throughout. The main function of AC is to reduce friction, to help the synovial fluid lubricate the joint, and to make it easier to distribute loads and forces across the bone beneath in a low-friction way [15]. AC consists of two principal phases: (1) a solid phase (collagen, Glycosaminoglycans (proteoglycans), chondrocytes and (2) a fluid phase (water, electrolytes, and nutrients). The constituents of the AC behave differently in the various zones classified as the superficial zone, the middle zone, the deep zone and the calcified zone with each zone marked by three distinct regions including the pericellular region, the territorial region and the interterritorial region (Fig. 1) [[9], [11], [16]]. Each of these zones exhibits specific structural, functional, and mechanical properties. Depending on their location in different zones chondrocytes respond specifically to different stimuli through the secretion of different proteins.

Multiple risk factors contributing to the development of OA include age, obesity, diet, bone density, joint malalignment, trauma, occupational influences, gender and ethnicity, genetic predisposition, biomechanics and abnormal loading, immobilization, etc. Inflammation [17], [18], [19], [20], [21], [22], [23], [24] and matrix metalloproteinases [25], [26], [27], [28] play a pivotal role in perpetuating the breakdown of cartilage in joints and promoting the progression of OA.

In this review, we present current scientific knowledge of OA and the pathogenesis of OA and we focus on the role of chondrocytes in cartilage formation and the progression of OA.

The current knowledge on the interplay of inflammatory and anti-inflammatory cytokines in the pathogenesis of OA is correlated with the activation of catabolic pathways, inhibition of matrix synthesis, and promotion of cellular apoptosis. The role of synovial tissue in promoting inflammatory and destructive responses in OA is presented here. The members of the metalloproteinase family are introduced and the structure and function of the MMPs are described highlighting their significance in the physiology and pathology of OA considering the mechanism of matrix degradation. Signal transduction pathways and MMP inhibitors are also presented.

## Role of inflammation in the pathogenesis of OA

The disorder of cartilage homeostasis is not the only cause of OA, rather OA involves the entire joint structure including the subchondral bone, synovial membrane, meniscus, ligaments, and periarticular muscles. Inflammation is regarded as a homeostatic response to the inactivation and destruction of invading pathogens and for the removal of waste and debris re-establishing normal function through resolution or repair [29]. Inflammation is classified as acute or chronic considering its duration [[29], [30], [31], [32]]. Changes in small blood vessels in acute inflammation cause accumulation of fluid and granulocytic cells at the site of injury producing a systematic response such as fever, leucocytosis, protein catabolism, and altered hepatic synthesis of plasma proteins. Resolution follows tissue destruction in acute inflammation whereas damage and repair take place simultaneously in chronic inflammation. After resolution tissue structure becomes normal but after repair organ changes functionally and morphologically. The inflammatory response is acute initially but does not necessarily turn into chronic inflammation and chronic inflammation does not precede acute inflammation. Tissue infiltration by macrophages and lymphocytes distinguishes chronic inflammation [21,33]. Mounting pieces of evidence have shown clear signs of inflammation along with the progress of structural changes in OA upholding the importance of proinflammatory mediators in the pathogenesis of OA [[18], [19], [22], [34], [35], [36], [37], [38]]. A possible interdependence of synovitis, OA inflammation, and the development of structural changes is demonstrated by these studies. Recent studies indicate that inflammation in OA develops from the interaction of the innate immune system and inflammatory mediators involving local tissue damage and metabolic dysfunction. A driving role is played by the early cartilage degradation in the development of inflammation within the joint especially in the OA synovium.

Synovium (SM), a specialized connective tissue, lines diarthrodial joints, encircles tendons, and creates the lining of bursae and fat pads [39,40]. The SM functions as a semipermeable membrane that regulates molecular traffic into and out of the joint area while retaining the composition of synovial fluid, which is crucial for maintaining the normal physiological condition of articular cartilage [41]. The synovial fluid volume and composition are maintained by SM through the production of lubricin and hyaluronic acid. Synovial fluid nourishes chondrocyte (together with subchondral bone) through diffusion and lubricates articular cartilage. There are two layers in a typical synovium. The outer layer, or subintima, is made up of several different types of connective tissues, including fibrous, adipose, and areolar. This layer is largely acellular and contains a significant amount of type I collagen, a microvascular blood supply, lymphatic vessels, and nerve fibers. A layer of 1–4 cells makes up the inner layer, or intima, which is located adjacent to the joint cavity. These synoviocytes are macrophages and fibroblasts, the latter being the dominant cell type in healthy SM. SM regulates the blood plasma ultrafiltrate from which the synovial fluid is produced.

Synovitis or synovial fluid inflammation is the situation when the synovium of a joint becomes inflamed (swollen) [42], [43], [44], [45]. Synovitis is marked by joint pain particularly in motion, stiffness, redness, warmth, and swelling, due to effusion (synovial fluid collection) in a synovial sac. With disease duration and related metabolic and structural changes in joint tissues, the synovial response pattern changes. The inflammatory alterations involve synovial hypertrophy and hyperplasia along with an enhanced number of lining cells and permeation of the subliming tissue with inflammatory cells. The inflamed synovial membrane finally results in the degeneration of cartilage and bone in the joint. An increase in macrophage infiltration and histological degree of inflammation enhances the level of synovial angiogenesis. The most widely accepted theory to explain the inflammation in OA is that when degraded cartilage fragments come into touch with the synovium, the synoviocytes perceive them as foreign particles and respond with a protective inflammatory response releasing pro-inflammatory mediators that attract cells, increase angiogenesis, and give rise to the phenotypic changes of chondrocytes. The by-products of cartilage injury and the stress response are factors that can cause joint inflammation, and obesity-related systemic inflammation may exacerbate local inflammation [43], [44], [45].

The inflammatory environment of osteoarthritis synovial fibroblasts (SFs) from the synovial tissue of the hand, hip, knee, and foot is influenced by obesity, joint stress, and anatomical location separately [46], [47], [48], [49]. There is a considerable degree of heterogeneity between patients who are obese and those who are normal weight due to the existence and preponderance of particular SF subsets, which are characterized by four functional molecular endotypes confirmed by bulk RNAseq and single cell RNAseq [47]. Obese patients have higher SF proliferative activity, which depends on the anatomical location. Inflammatory SF subgroups in obese patients are localized in the subliming and lining layers of the OA synovium, and can be identified by the differential expression of transcriptional regulators FOS and MYC. The transcriptome phenotype of SFs from load-bearing and non-load-bearing joints is significantly impacted by obesity.

Differential phenotypes of synovial tissue and separate populations of synovial fibroblasts are seen at the sites of patient-reported pain in knee OA. Various synovial fibroblast subgroups and a differential transcriptome phenotype are observed in synovial tissue obtained from patient-reported pain locations in individuals with early and end-stage OA. Functional pathway analysis demonstrates that synovial tissue and fibroblast subsets from painful locations stimulate inflammation, fibrosis, and neuronal development and activity.

Notably, subsets of fibroblasts from the painful areas of early-stage OA patients show gene signatures that support nociceptive signaling pathways, fibrosis, inflammation, and neuronal development. Long noncoding RNAs (lncRNAs), specifically MALAT1, are key regulators of the inflammatory response in the OA synovial joint by showing that obesity in OA patients is linked to an inflammatory synovial fibroblast phenotype. However, the proinflammatory cytokine IL-6 is secreted by synovial fibroblasts from obese OA patients in higher amounts than those from normal-weight patients [49]. This is consistent with the higher levels of IL-6 identified in the synovial fluid of obese OA patients. IL-6 produced from chondrocytes stimulates the release of IL-6 from synovial fibroblasts. Obesity increases the pro-inflammatory adipokine and leptin levels, which in turn increases the cross-talk between chondrocytes and synovial fibroblasts and increases IL-6 production in OA patients. Over time, higher synovial inflammation and faster cartilage degradation are brought on by more cytokines and proteolytic enzymes. Pro- and anti-inflammatory mediators are produced by synovial cells in response to clinical factors such as trauma, age, obesity, and excessive mechanical stress.

Menisci are crescent-shaped wedges of fibrocartilaginous tissue that function to diminish friction and distribute loads. In joints, a critical protective role is played by the meniscus in maintaining the stability of the joint, absorbing shocks, reducing friction, and transferring and distributing mechanical load between the femoral condyles and tibial plateaux with a contribution to proprioception [50], [51], [52]. Fibrillation and disruption are observed to take place first in the rim spreading later to the articular surfaces of the meniscus and finally leading to total damage or loss of meniscus tissue. Severe matrix disarrangement occurs in case of moderate or severe OA in the meniscus with unusual cell clustering. When articular cartilage experiences abnormal biomechanical forces the meniscal disruption may intensify inflammation. Growth of blood vessels and perivascular nerves leads to increased meniscal vascular density in OA patients.

The development of new blood vessels, or angiogenesis, is crucial to the pathophysiology of OA. Angiogenesis regulates chondrocyte function, promotes chondrocyte hypertrophy, gives rise to abnormal tissue growth and perfusion, and endochondral ossification [[34], [53], [54]]. Angiogenesis is upregulated in the synovium, menisci, and osteophytes during OA, which causes ossification in osteophytes and the deep layers of articular cartilage. In osteoarthritic joints, sensory nerves grow along newly formed blood vessels and they eventually penetrate noncalcified articular cartilage, osteophytes, and the inner regions of menisci. Angiogenesis contributes to synovitis, osteochondral damage, and meniscal pathology in patients with OA. In OA, angiogenesis and inflammation are strongly associated processes that may have an impact on pain and the progression of the disease. In comparison to healthy joints, the level of oxygen in articular chondrocytes of inflammatory joints is comparatively lower. Because of their insufficient vascular supply of oxygen to the reserve and hypertrophic zones, low oxygen tension, or hypoxia, is a major stimulator for angiogenesis. More specifically, decreased oxygen tension activates a transcription factor called HIF-1α, which in turn activates vascular endothelial growth factor (VEGF) expression [55].

Vascularization is accompanied by innervations, and mechanical overload, hypoxia, and inflammation can activate these newly formed nerves causing enhanced pain that persists even when inflammation is reduced. Proangiogenic factors may also promote nerve growth, and vascular cell-produced compounds may direct and stimulate neuron growth [56]. Common pathways connecting blood vessels and nerve growth involve the release of proangiogenic substances like neuropeptides, β-nerve growth factor, VEGF, angiopoietins, HIFs, proinflammatory cytokines, different chemokines, matrix elements, cell adhesion molecules, proteases and other agents. The balance between angiogenic and anti-angiogenic factors in the joint controls blood vessel growth. Angiogenesis can be stimulated directly or indirectly by inflammatory mediators which include the mast cells and macrophages that are abundant in chronically inflamed osteoarthritic synovium. Factors released by macrophages have the direct ability to trigger new vessel development. Macrophages possess the ability to emit substances that induce other cells, including fibroblasts and endothelial cells, to generate angiogenic factors like VEGF. Lymphocytes and neutrophils may produce angiogenesis-promoting molecules including VEGF and basic fibroblast growth factor (bFGF), and may play a role in the early induction of angiogenesis [[34], [53], [56]].

## Role of mediators in OA inflammation

**Fig. 2:**
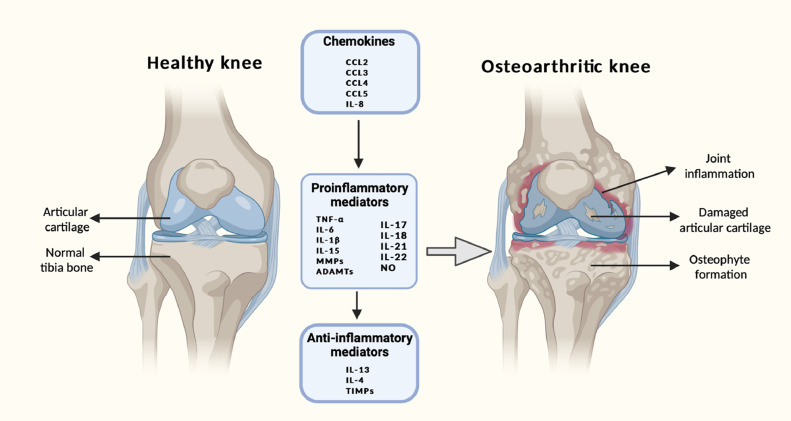
Key inflammatory mechanisms and causes of osteoarthritis are represented schematically. The secretion of enzymes and other inflammatory factors involved in the pathogenesis of osteoarthritis, which results in morphological changes within the joint like cartilage degeneration, osteophyte formation, and other inflammatory changes like synovitis, is caused by the disturbed balance of proinflammatory and anti-inflammatory cytokines (in favor of proinflammatory cytokines). Inflammatory processes are further aided by chemokines, which promote the chemotaxis of inflammatory cells, which in turn generate proinflammatory cytokines. This vicious cycle makes treating and stopping the progression of osteoarthritis extremely difficult. MMPs, interleukin (IL), chemokine ligand (CCL-CC), TNF, ADAMTS, COX-2, PGE-2, and NO.

The processes taking place inside the joint involve both inflammatory catabolic and anti-inflammatory anabolic processes occurring continuously, driven by different mediators. A vital role is played by interactions inside the cytokine network. Metabolic homeostasis of tissues in the joint is lost to a great extent due to the inflammatory cytokines through the promotion of catabolic and destructive processes. The dysregulation of the cytokine network consisting of inflammatory and anti-inflammatory cytokines is manifested in the imbalance of metabolism detected in OA [[18], [19], [20], [22], [24], [57]]. The most important inflammatory cytokines involve IL-1β, TNFα, IL-6, IL-15, IL-17, and IL-18 and anti-inflammatory cytokines include IL-4, IL-10, and IL-13, IFN-γ with regulatory cytokines IL-6 (negative regulator of chondrocyte proliferation), IL-8 (regulator of chondrocyte hypertrophy). Standard biomarkers of inflammation also include chemokines, adipokines, and collagen derivatives of nitrous oxide (Fig. 2)[58]. The inflammatory cytokines increase the synthesis and release of many proteolytic enzymes including MMPs and ADAMTS which contribute to the decomposition of articular cartilage by their effect on the chondrocytes, synovial cells, and other articular, and periarticular tissues. In addition to this, the cells of the immune system that migrate to the site of inflammation are affected by these enzymes. The duration and severity of the disease help to vary the production and operation of different cytokines during the progression of OA [59]. Inflammatory prostaglandin E2 (PGE2), cyclooxygenase-2 (COX-2), phospholipase A2 PLA2, NO, and free radicals are produced by these cells under the influence of the inflammatory cytokines. Anti-inflammatory cytokines inhibit the production of inflammatory cytokines especially IL-1β and TNFα along with an increase in proteoglycan synthesis, inhibition of apoptosis of chondrocytes, decrease of synthesis and secretion of metalloproteinases and the degradation of the level of PGE2. Anti-inflammatory cytokines act mainly on the cells stimulated by inflammatory cytokines without any significant difference with the metabolism of cells not stimulated in this way.

### Proinflammatory mediators

**(1) Interleukin-1 Beta (IL-1**β**)**

IL-1 is the group of inflammatory cytokines that are vital signaling molecules to mediate inflammation in the immunological response to a broad spectrum of stimuli in both innate and adaptive immune systems [60,61]. There are two distinct forms in (IL -1) family encoded by different genes: IL-1α and IL-1β. The synovial fluid, synovial tissue, cartilage, and the subchondral bone layer of OA patients are observed to contain an upgraded level of IL-1β. IL-1β is produced by the macrophage, and assists lymphocytes in fighting infections and migrating through blood vessels to the sites of infections. Chondrocyte, osteoblasts, cells in the synovium tissue, and mononuclear cells controls the synthesis of IL-1 β. Interleukin-1β is a potent pro-inflammatory cytokine performing in the pathogenesis of OA. Inflammatory reactions and catabolic effects are induced by IL-1β in combination with other mediators in articular cartilage and other constituents of the joint. It is a cytokine protein encoded by the IL-1 B gene and is produced as a cytosolic precursor protein (pro-IL-1β) which comprises 269 amino acid residues [62]. The inactive pro-IL-1β is produced in response to pathogen-associated molecular patterns (PAMPs).

The active configuration of IL-1β consists of 153 amino acid residues and is produced by activated macrophages as a proprotein through proteolysis regulated by the enzyme Caspase 1/IL-1β converting enzyme, (CASP1/ICE) and is released into the extracellular space [63]. Inflammatory pain hypersensitivity is contributed by this cytokine in the central nervous system inducing COX-2 (PTGS2/COX2). This cytokine takes part in various cellular activities such as proliferation, differentiation, and apoptosis. Signal initiation occurs by a stepwise method in which an agonist cytokine attaches with a cognate receptor with the subsequent recruitment of a secondary receptor by the cytokine-receptor complex. IL-1β activates cells biologically interacting with TLR such as the IL-1R1 activated by PAMPs or cytokine signaling [64].

Other receptors IL-1R2 can bind IL-1β and produce an inactive ligand-receptor complex after binding with IL-1β exhibiting incapability of communication and activation of the intercellular signal [65], [66], [67]. The receptor antagonist IL-1Ra is produced by some cells of the joint and can link with IL-1R1 and IL-1R2. This bonding blocks their linkage with IL-1β thereby affecting the reduction of the activity of IL-1β to some extent [68,69]. In OA patients there is an enhanced expression of IL-1R1 on the surface of chondrocytes and fibroblast-like synoviocytes (FLS) [70,71]. After the linkage of IL-1β with TLR receptors, for example, IL-1R1, an additional IL-1R3 chain (IL-1RAcP) is recruited to grow a complex by the intracellular Toll-IL-1R resulting in the recruitment of adapter protein MyD88 [72]. IL-1β obstructs chondrocytes in the synthesis of ECM and the synthesis of structural proteins - type II collagen and aggrecan is also interfered with. The performance of chondrocytes is affected by the synthesis of enzymes of the metalloproteinases group which can destroy the constituents of articular cartilage. Production of ADAMTS by chondrocytes is also affected by IL-1β whereas production of ADAMTS-4 is stimulated by IL-1β and TNF [73]. Under the effect of IL-1β and TNFα aging and apoptosis are induced in the chondrocytes with inhibition of restoration, and enhancement of the damaging effect of enzymes. In an autocrine process, IL-1β can induce its synthesis and trigger the synthesis of other cytokines [74], [75], [76], [77]. The overexpression of IL-1R1 in the cartilage of OA joints is observed to increase the binding of IL-1β which is responsible for the catabolic events present in OA.

**(2) Tumour Necrosis Factor Alpha (TNFα)**

TNFα is one of the most important inflammatory cytokines created by macrophages or monocytes in the course of acute inflammation [[19], [58], [78]]. TNFα belongs TNF superfamily containing 19 ligands [79]. Human TNF-α is a 17 kDa cytokine consisting of 157 amino acids with a 76 amino acid pre-sequence and can exist in soluble form or unprocessed membrane-bound form with 26 kDa, and 233 amino acids. As TNF is translocated to the cell membrane, the membrane-bound 26 kDa molecule is activated by the TNF-converting enzyme and released into the extracellular environment as a 17 kDa protein. It is secreted not only by immune cells such as mononuclear cells but also by chondrocytes and osteoblasts. OA joints show an increase in the amount of TNFα in synovial fluid and membrane. A wide range of signaling phenomena is regulated by TNFα giving rise to necrosis and apoptosis. The inflammatory cascade is driven by TNF α and catabolic events are induced by the increased expression of TNF-α increasing MMPs. The synthesis of main ECM components is downregulated by TNF α through inhibition of anabolic activities of chondrocytes and reduction of the production of type II collagen. In chondrocytes, the production of MMP-1, −3, and −13 is induced and the production of aggrecanases (ADAMTS)−4 and −5 is stimulated by the combined expression of IL-1β and TNF α. There is overexpression of TNF receptor type I (TNFRI) in chondrocytes of OA patients. The arthritic condition is enhanced by IL-1β and TNF α through the induction of the production of proinflammatory cytokines, (such as IL-6, IL-8, and monocyte chemoattractant protein). The secretion of TNFα and IL-1β and their increased concentration are observed in synovial fluid, synovial tissue, cartilage, and subchondral bone layer. Two isotypic membrane receptors TNF-R1 and TNF-R2 are located on the surface of almost every nucleated cell and the cytokine can bind them [80,81]. Soluble and membrane forms can activate the TNF-R1 receptor whereas the membrane form mainly attaches to TNF-R2. TNF-R1 has a greater impact on the destruction of articular cartilage than TNF-R2 though after the activation by TNFα both of them participate in the signal transduction processes in OA. Increased expression of TNF-R1 is observed in fibroblast-like synoviocytes (FLS) cells [82,83]. Under the influence of TNFα chondrocytes produce in large amounts NO, COX-2, and PGE2 which cause the enhancement of MMP activity, inhibition of the production of anabolic products (collagen and proteoglycan) and induction of chondrocyte apoptosis leading to inflammation and articular cartilage destruction. The development of OA is determined in advance by the polymorphism of the gene (M196R) encoding the receptor protein TNF-R2 by multiplying the number of receptor proteins on the surface of the chondrocytes and as a result, their activity is disrupted by the overactivation by mTNF [84]. Polymorphism in the TNFα ligand facilitates the progression of OA. Additional ligands with anti-inflammatory and immunomodulatory properties such as progranulin (PGRN), PC-cell derived growth factor (PCDGF), proepithelin (PEPI), or acrogranin can incorporate both TNF-R1 and TNF-R2. A significant increased level of PGRN is observed in OA patients. PGRN with its ability to link with TNFα and its enhanced level in the progress of OA intervenes in the signaling pathway TNFα/TNF-R1 and TNFα/TNF-R2 antagonistically. Therefore, the development of OA can be accelerated as well as inhibited by the imbalance of TNFα/PGRN [85,86].

**(3) Interleukin-15 (IL-15)**

IL-15 is a glycoprotein with the configuration of interconnected α-helices with a mass of 14–15 kDa [[80], [81], [82], [83], [84],]. It stimulates and enhances the differentiation, proliferation, and maintenance of T cells, NK cells, and CD8+ memory cells [87]. In the early stage of OA synovial fluid exhibits an elevated level of concentration of IL-15. This enhanced level of concentration correlates with the sensation of pain and the severity of lesions [88]. It is also observed that it can induce the secretion of metalloproteinases from the MMPs group . Complexes created by the interaction of IL-15 with cell surface IL-15 Rα react with complexes produced by the interaction of IL-2 Rβ and the common gamma chain on adjoining cells. By this trans-presentation mechanism cells respond to IL-15 even in the absence of IL-15 Rα. Reverse signaling is induced by the ligation of membrane-associated IL-15/IL-15 Rα complexes thereby promoting the activity of the IL-15/IL-15 Rα expressing cells.

**(4) Interleukin-17 (IL-17)**

The IL-17 family of cytokines is a class of cytokines identified to play a potential inflammatory role in the pathogenesis of OA. IL-17 is a family of six cytokines (IL-17A-F) with five types of receptors (IL-17RA-E) [[89], [90], [91], [92], [93], [94]]. IL-17 is mainly secreted by IL-17-producing T helper (Th17) cells, stimulated C4+ T cells, and mast cells that are observed as cellular infiltrates through the blood vessels into the D synovial membrane and the OA joint. Chondrocytes and fibroblast-like synoviocytes are the main cells influenced by IL-17 and express IL-17 receptor (IL-17R) on their surface [[95], [96]]. An increased concentration of IL-17 is found in the serum and the synovial fluid from OA patients. IL-17 can inhibit proteoglycan synthesis by chondrocytes and affect ECM by enhancing the production of MMPs. In addition, it can affect cartilage by inducing the production of other tissue-degrading cytokines and compounds such as IL-1β, TNFα, IL-6, NO, and PGE2 [97]. The secretion of VEGF by chondrocytes and FLS is influenced by IL-17 and causes hypertrophy of the synovial membrane through excessive blood vessel formation. The polymorphism in the gene IL-17A G-197A can be correlated with the susceptibility to the development of OA. Furthermore, in the cells of end-stage OA patients, IL-17A is observed to grow increased gene or protein expression of inflammatory mediators such as IL-6, IL-8, leptin, resistin, CCL7, NGF, CXCL1, CCL2, COX2, and iNOS, thereby describing a potential inflammatory OA phenotype[,]. The heteromeric receptor complex, consisting of IL-17 receptor A (IL-17RA) and IL-17RC is used by IL-17A, IL-17F, and IL-17AF to transmit signals.

**(5) Interleukin-18 (IL-18)**

IL-18, a cytokine originally identified as an interferon-γ (IFN-γ)-inducing factor, has a structure similar to that of IL-1 and is acknowledged as a member of the IL-1 superfamily of proteins [[98], [99], [100]]. It is synthesized as a biologically inactive precursor form of pro-IL-18, comprising 192 amino acid residues, which is converted into a biologically active form by the activation of Caspase 1 or proteinase 3, consisting of 157 amino acid residues [[101], [102], [103], [104]]. An elevated level of Caspase 1 is observed in the articular cartilage and synovium in OA resulting in the promotion of IL-18 and IL-1β [105]. A functional 18 kDa form is produced by being cleaved by the IL-1 converting enzyme (ICE, also known as caspase I). Chondrocytes, osteoblasts, FLS, and macrophages regulate the production of IL-18 [[106], [107]]. IL-18 is found with a large concentration in the synovial fluid, synovium, cartilage, and blood serum establishing a strong correlation with the severity of OA [108]. IL-18Rα receptor mediates the effect of IL-18 and the signal is transmitted by IL-18 in the presence of IL-18Rα recruiting the IL-18Rβ (IL-18RAcP) chain [109]. IL-18 affects chondrocytes through the upregulation of IL-18Rα on the surface of chondrocytes and stimulates the excessive production of MMP-1, MMP-3, and MMP-13. The production of proteoglycans, aggrecan, and type II collagen is inhibited under the influence of IL-18 and chondrocytes undergo morphological changes similar to apoptotic processes. Chondrocytes and synovial cells are affected by IL-18 through the increase in the synthesis of a range of compounds and enzymes such as IL-6, iNOS, PGE2, COX-2, and VEGF [[106], [110], [111]].

### Anti-Inflammatory cytokines

IL-4, IL-10, and IL-13 are the main anti-inflammatory cytokines taking part in the origination and development of OA and represent a larger group of anti-inflammatory cytokines. Cytokines are observed to decrease the production of IL-1β and TNFα and upregulate the production of IL-1 receptor antagonist IL-1Rα and TIMP-1, as well as inhibition of PGE2 release in synovial fluid of OA patients. suggesting the potentiality of these anti-inflammatory cytokines in the therapeutic use in OA. Several catabolic pathways associated with OA progression can be blocked by IL-1Rα, a competitive inhibitor of IL-1R. These pathways involve PGE2 synthesis, collagenase and NO production by chondrocytes, and cartilage matrix degradation.

**(1) Interleukin-4 (IL-4)**

IL-4 is constituted of 129 amino acids forming a compact, globular structure with a four alpha-helix bundle stabilized by three disulfide bonds [[112], [113], [114], [115], [116], [117]]. The receptors for IL-4 are heterodimeric complexes composed of the common gammachain and a special cytokine-specific subunit. IL-4 signaling is mediated through the IL-4 receptor α-chain (IL-4Rα) existing in the form of two different complexes composed of two or three receptor chains, such as IL-4Rα, IL-13Rα1, and IL-2Rγc [[118], [119], [120]]. Type 1 signaling complex, located mainly on hematopoietic cells, is produced through the dimerization with the common gamma chain of IL-4Rα and IL-2Rγc and facilitates the bonding of IL-4 [121]. The type 2 complex is expressed on non-hematopoietic cells and is composed of IL-4Rα and IL-13Rα1 with the ability to attach to IL-4 and IL-13 whose biological activities are closely related [122]. The development of OA in the joints is predetermined by the polymorphism of the genes encoding IL-4 and IL-4Rα [123]. IL-4 is produced by helper T cells (Th2 type) in response to the participation of the T cell receptor and also by mast cells and basophils through cross-linking of the receptor for immunoglobulin E (IgE) [96]. An elevated level of soluble IL-4Rα (sIL-4R) is detected in the serum of OA patients and the concentration of IL-4 is observed to increase in the synovial fluid and synovial cells . IL-4 possesses a chondroprotective property as it can inhibit the degradation of proteoglycans, and reduce the production of MMPs [124]. During the progress of OA, chondrocytes exhibit decreased susceptibility to the protective effect of IL-4 causing rapid degeneration of articular cartilage under the influence of proinflammatory cytokines [125]. It is also observed that IL-4 alone or in combination with IL-10 can inhibit the apoptosis of the chondrocytes and FLS and reduce the production of various inflammatory mediators including IL-1β, TNFα, and IL-6, PGE2, COX-2, PLA2, and iNOS along with the upregulation of the expression of TNFα receptors [124].

**(2) Interleukin-10 (IL-10)**

IL-10 is a helical cytokine that structurally belongs to the class II cytokine family including IL-19, IL-20, IL-22, IL-24, IL-26, and interferons (IFN-α, -β, and -γ) [117]. It is a non-covalent homodimer where each monomer is a polypeptide chain comprised of 160 amino acid residues. Its structure consists of six interconnected α-helices (A-F) with a molecular mass of 37 kDa (18.5 kDa for each one) [126]. IL-10 acts as an anti-inflammatory cytokine by stopping the growth and activation of Th1 cells and Th17 and elevating the development of M2 macrophages and regulatory T cells. Initially, IL-10 binds to the IL-10 receptor (IL-10R) which is a member of the class II cytokine receptor family and composed of two subunits, IL-10R1 and IL-10R2 [[117], [120], [127], [128]]. At first, the receptor is activated by the linkage of IL-10 to the IL-10R1 subunit with high affinity, then a conformation change takes place in such a way that the IL-10 and IL-10R1 complex can bind to the IL-10R2 subunit. By decreasing TNF-α production and TNF-α mediated events related to OA development, IL-10 inhibits the effect of TNF-α on synovial fibroblasts of OA patients, and a significant reduction in the production of PAGE2, COX-2, and PLA2 was also observed [[129], [130]].

**(3) Interleukin-13 (IL-13)**

IL-13 is a protein encoded by the IL13 gene. It has a mass of 13 kDa and a structure of four interconnected α-helices spanning helix A, helix B, helix C, and helix D with an up-up-down-down topology that involves a β-sheet created between residues in the AB-loop and CD-loop [[131], [132]]. IL-13 has a close similarity to IL-4 with a 25 % sequence identity containing two of the three disulfides present in IL-4. IL-13 is a pleiotropic T-helper type 2 (Th2) cytokine that induces both pro-inflammatory and anti-inflammatory immune responses [133]. A receptor system made of the combination of IL-4 and IL-13 is required to mediate the action of IL-13 as a ligand. For both the IL-4 and IL-13 receptors, the α-chain of the IL-4 receptor (IL-4Rα) performs as the principal signaling chain. The IL-13 receptor is constituted of at least one of two IL-13 binding chains, IL-13Rα1 or IL-13Rα2 [[122], [134]]. IL-13-induced signal transduction is initiated by the linkage of IL-13 to IL-13Rα1 through the recruitment of the α-chain of IL-4 receptor (IL-4Rα) thereby producing a type 2 heterodimeric receptor complex. It is worth mentioning that the relatively low affinity of IL-13 for IL13Rα1 is enhanced approximately ten times in the presence of IL4Rα [[135], [136]].

IL-13 is reported to be anti-inflammatory and chondroprotective on AC and synovium in OA. IL-13 cytokines are produced by macrophages, monocytes, T-lymphocytes, B cells, mast cells, NK cells, and endothelial cells, and promote and control anti-inflammatory responses by the inhibition of the production of inflammatory cytokines.

Both IL-13 and IL-4 decrease PGE2 production stimulated by IL-1α by inhibiting the synthesis of COX-2 but do not affect the level of synthesis of PLA. The results demonstrate that the nuclear concentration of transcription factors C/EBP is decreased by IL-13 thereby affecting the inhibition of the synthesis of COX-2. These results uphold the potentiality of the clinical utility of IL-13 in the treatment of OA to protect chondrocytes by blocking the inflammatory processes through the reduction of the secretion of inflammatory cytokines and metalloproteinases.

## Reactive oxygen species (ROS) and OA

**Fig. 3:**
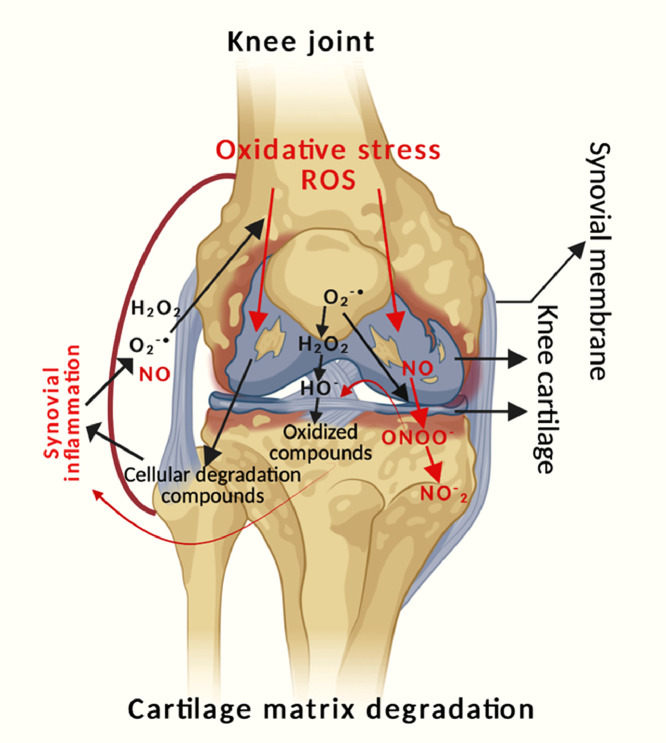
**Interdependence relationship between oxidative stress and inflammation in osteoarthritis**.

A major regulator of the angiogenic response to inflammation appears to be the balance between NO and ROS generation. ROS can cause essential chondrocyte death and matrix breakdown, which can contribute to the start and progression of OA. The hydroxyl radical (OH-), H_2_O_2_, superoxide anion (O_2_-), NO, and hypochlorite ion (OCl-) are examples of ROS, which are free radicals that include oxygen molecules (Fig. 3) [[137], [138], [139]]. ROS are short-lived, unstable, and highly reactive due to the presence of unpaired electrons in the valence shell. The mitochondria (via oxidative phosphorylation), nicotinamide adenine dinucleotide phosphate (NADPH) oxidase, and xanthine oxidase (XO) are the primary sources of ROS formation. Of these, mitochondria are the most likely source and only around 2–3 % of the total O_2_ utilized by active mitochondrial electron transport chains is incompletely converted to O_2_^−^, rather than to water. The main ROS producers in phagocytes are the NADPH oxidase enzymes (NOXes), which produce ROS via the reaction 2O_2_+ NADPH→2O_2_- + NADP^+^+ *H*^+^. Damage to macromolecules and disruption of redox signaling and regulation are the effects of oxidative stress, which is defined as an imbalance between the generation of ROS and antioxidant defenses.

### Nitric oxide (NO) and OA

**Fig. 4:**
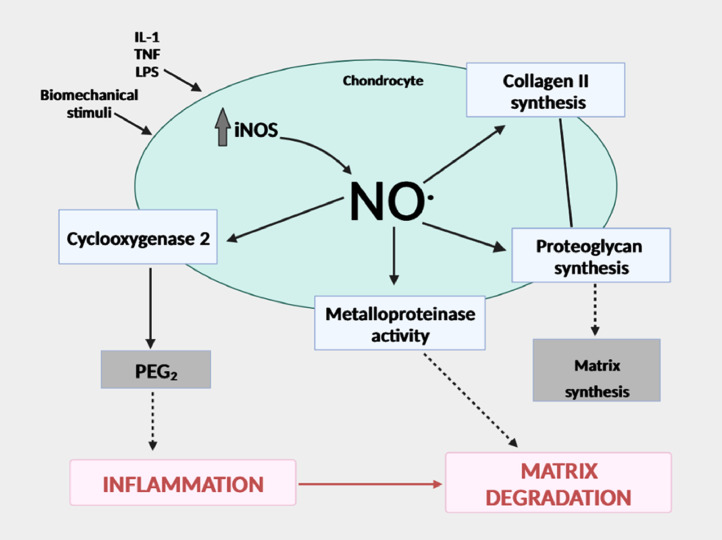
NO involved in the etiopathogenesis of osteoarthritis.

NO, a signaling molecule, plays a key role in the pathogenesis of inflammation and performs an anti-inflammatory role under normal physiological conditions [[140], [141]]. On the contrary, NO is regarded as a pro-inflammatory mediator that, when overproduced under abnormal circumstances, causes inflammation. By damaging the host tissues and the invading microorganisms, excessive NO generation in chondrocytes causes cartilage destruction and cellular damage. NO can easily interact with (O_2_-) to create peroxynitrite, which can nitrate certain proteins and peroxynitrite can develop in AC [[141], [142]]. NO is the major pro-inflammatory mediator in OA. Numerous immunological and inflammatory cell types, including macrophages, T lymphocytes, antigen-presenting cells, mast cells, neutrophils, and NK cells, are regulated in terms of their functional activity, growth, and death. Through the activity of iNOS, which is present in both invading leucocytes and activated, resident tissue cells, large amounts of NOs are created at sites of inflammation. NO, which is necessary for healthy physiological homeostasis, is produced in small amounts by constitutively expressed NOS. However, as an initial anti-inflammatory response, inflammatory stimuli including endotoxins, cytokines, and growth factors promote the production of iNOS and catalyze a high NO output. NO is an endogenous free radical that is created when l-arginine is converted to l-citrulline, with oxygen acting as an electron acceptor and NADPH serving as the electron donor [141]. NOSs are a family of enzymes that catalyze the production of NO and exist in three different isoforms such as eNOS (endothelial NOS or NOS_1_), iNOS (NOS_2_), and nNOS (neuronal NOS or NOS_3_). Endothelial cells and neurons provided the initial evidence for eNOS and nNOS, respectively but later studies also demonstrated their presence in other cell types. LPS and inflammatory cytokines like IL-1, TNF, or IFN stimulate NOS_2_, which is only expressed in activated cells while NOS_1_ and NOS_3_ are expressed constitutively [[143], [144], [145]]. NO is produced by converting l-arginine to NOH-arginine and then to l-citrulline with NO (Fig. 4) [146]. NOS_2_ catalyzes the reaction of NO synthesis by oxidizing one of the guanidinyl nitrogens of arginine to anhydroxy arginine, which is then further oxidized to citrulline and NO. After many hours of stimulation, NO production by NOS_2_ is induced; however, once induced, it can continue for up to five days. The ability of NO to interact with superoxide anions (O_2_-) to produce ROS is correlated with its damaging effects. Peroxynitrite (ONOO-), a potentially more harmful product, is created when the unpaired radical electrons on NO and O_2_ combine [[147], [148]]. S-nitrosothiols, which have roles in signaling and modulating cellular and enzyme activity, and are critical regulators of physiology and pathology, can also be formed when NO binds to a reactive cysteine thiol. NO decreases IL-6 synthesis by chondrocytes and Kupffer cells as well as γ-IFN and TNF-α production by splenocytes. There is evidence to suggest that the effects of NO may vary depending on time and/or concentration. NO appears to have pro- and anti-inflammatory capabilities in the joint, depending on its concentration and cellular source. Furthermore, researches indicate that reactive oxygen species and NO derivatives of (ROS) may also produce opposite results, both destructive and protective. In multiple cell types the protective roles of NO in addition to the conflicting behaviors in cultured chondrocytes, upholds the possibility that NO may have additional protective effects in the function of chondrocytes. By mediating the generation of proinflammatory cytokines, limiting the synthesis of collagen and proteoglycans, being involved in the destruction of MMPs, and inducing apoptosis, NO plays a catabolic function in the development of OA [149]. Recent research, however, raises the possibility that NO and its redox by-products may help protect cartilage. NO and its derivatives also play crucial roles in both the generation and suppression of nociception and pain which are the main factors contributing to functional impairment in OA [150].

## The role of MMPs in osteoarthritis

MMPs play a role in a variety of physiological and pathological processes in their normal state, but different clinical disorders result from changes in MMPs [[151], [152], [153], [154], [155], [156], [157]]. MMPs, a family of at least 28 structurally related zinc-dependent proteolytic enzymes, can degrade all of the extracellular matrix's constituents, including collagen, laminin, fibronectin, vitronectin, and proteoglycans. This degradation of ECM through cleavage of internal peptide bonds of the target proteins is the hallmark of joint arthritis. When chondrocytes and synoviocytes are subjected to severe mechanical stress and injury in joints, the release of pro-inflammatory cytokines such as IL-1β, TNF-α, and TGF-β promotes MMP expression [[147], [148], [158], [159], [160], [161], [162], [163], [164]]. MMPs are released as inactive proenzymes (Zymogen) and play critical roles in normal physiological processes including development and wound healing. Their activation is turned on at the tissue level through the degradation of the N-terminus of the propeptide by other proteases. MMPs express their catalytic activity by binding themselves to membrane receptors and overexpression of MMPs is observed in case of different pathological conditions such as irreversible tissue destruction and collagen degeneration in OA. The expression of MMPs in normal joints is augmented in arthritic tissues [25,28,154,[165], [166], [167], [168], [169], [170], [171], [172]].

The amino acid sequences of MMPs are comprised of two conserved domains for example a catalytic domain, and a prodomain for their substrate specificity. The substrates of the MMP family involve gelatinases (MMP-2, MMP-9), collagenases (MMP-1, MMP-13), matrilysins (MMP-7), stromelysins (MMP-3), membrane-type MMPs (MT-MMPs), and metalloelastase (MMP-12) [173]. The expression of MMPs is regulated for secretion, transcription, activation, and inhibition of the activated enzyme. Cells cannot express some MMPs which are rather expressed constitutively by signals like cytokines, growth factors, hormones, and modifications in cell-cell and cell-matrix interactions. MMPs are released by pro-inflammatory cells including fibroblasts, osteoblasts, endothelial cells, macrophages, lymphocytes, vascular smooth muscles, cytotrophoblasts and neutrophils [[26], [174]]. In the activation process, endopeptidases such as other MMPs, serine proteases, plasmin, or furin remove the pro-domain through the proteolytic attack in the bait region. The partial removal of the prodomain destabilizes the rest of the prodomain as the stepwise activation proceeds. The MMP activation is also induced by physiochemical agents like heat, low pH, chaotropic agents, and thiol-modifying agents disarranging the cysteine- Zn^2+^ coordination. Growth factors, inflammation, and oxidative stress are some examples of stimulating factors that trigger the generation of MMPs, which then result in the up-or-down-regulation of the MMPs with subsequent ECM remodeling. In most cases, MMPs' endogenous inhibitors, TIMPs, limit excessive MMP activation. The majority of MMPs, including MMP-1, MMP-2, MMP-3, MMP-8, MMP-9, MMP-10, MMP-13, and MMP-14, are connected with the turnover of ECM and the breakdown of AC in OA. But for this degradation to take place, the soluble collagenases MMP-1, MMP-8, and MMP-13 are essential, with MMP-13 predominating. Type II collagen, which is cleaved five times quicker than collagen I, six times faster than collagen III, and more readily than other collagenases, is the preferred substrate for MMP-13 [175]. MMP-13, which aggressively breaks down type II collagen, is thus specifically linked to the degeneration of AC in OA.

**(a) Structure of MMPs**

The 3D structures of MMPs have been determined by X-ray crystallography and NMR Spectroscopy. The MMPs have a common multi-domain structure consisting of the propeptide of about 80 amino acids, a catalytic domain of 170 amino acids, and a hemopexin (Hpx) domain of about 200 amino acids which is linked to the catalytic domain by a flexible hinge or linker region of variable lengths (∼75 amino acids) [[20], [23], [176], [177], [178], [179], [180], [181], [182], [183], [184]]. Initially, the MMPs are inactive zymogens containing a pro-peptide domain which is part of the “cysteine switch” [178,184]. The interaction of its cysteine residues with the zinc in the active site prevents the linkage of the substrate thereby giving rise to the inactivity of the enzyme. The propeptide domain is comprised of three α-helices with connecting loops. The first loop located between helix 1 and helix 2 is a protease-sensitive “bait region” [20]. The α (3)-helixes are followed by a “cysteine switch” which is a very conserved region with the sequence (PRCGXPD) and resides in the substrate binding site. The oblate spherical catalytic domain consists of three α-helixes, five β-sheets (S) (four parallel: β2–β1–β3–β5 and one anti-parallel: β4), connected by eight loops [23]. The first and longest β-sheet (β1) is located just after the initial loop and a second loop lies just before the large α-helix spreading longitudinally to the protein site. The short loop, loop 4, bridges the β-strands β2 and β3 and after β3 the sequence connects to an S-shaped double loop extending to the antiparallel β-strand β4 which is required for the attachment of peptide structure or inhibitors through the formation of hydrogen bond. Histidine residues are found in the conserved sequence HExxHxxGxxH, a zinc-binding motif. A flexible and proline-rich hinge or linker region connects the catalytic domain and the hemopexin domain. It has a variable length ranging from 14 to 75 amino acids. The hemopexin domain has an ellipsoidal disk shape made up of a four-bladed β-propeller structure with a single stabilizing disulfide bond between blades 1 and 4. Each blade consists of four antiparallel β strands and the four β-sheets possess similar scaffolds. Generally, one calcium ion and chloride are accommodated in the center of the propeller. β-Propeller structures present a large flat surface to facilitate interactions. This domain is essential for collagen triple helix degradation and substrate specificity and provide the site for interaction with TIMPs The substrate specificity [185,186] of MMPs depends on the substrate binding sites or pockets (S) within the MMP molecule located on both sides of the catalytic zinc atom (S1, S2, S3, … Sn localized on the left side; and S1´, S2´, S3´, … Sn’ localized on the right side) and these pockets interact with the substrate through the analogous substituents (P) in the substrate such as P1, P2, P3, . . . Pn and primed P1´, P2´, P3´, … Pn’ substituents, respectively [21,187,188,189,190].

**(b) The MMP family**

According to their structure and substrate specificity, MMPs are categorized into six classes, including membrane-type MMPs, collagenases, gelatinases, stromelysins, matrilysins, and others where MMP-14, −15, −16, −17, −24, and −26 belong to the membrane-type MMPs, MMP-1, −8,−13, and −18 fall in the collagenases category, MMP-3, −10, and 11 in the stromelysins, MMP-7 and −26 in the matrilysins and MMP-12, −19, −20, −21, −22, −23,−27 and −28 in others [20,23,[191], [192],^193^,^177^,^194^,[195], [196], [197]]. Interstitial collagens are broken down by collagenases, type IV collagen in the basement membrane is attacked by gelatinases, and non-collagen matrix proteins are broken down by stromelysins. Seven MMPs such as MMP-1, MMP-2, MMP-3, MMP-8, MMP-9, MMP-13, and MMP-14 are expressed at low levels in normal joint tissue but are systematically expressed at enhanced levels in AC under various circumstances. Out of these seven, four MMPs including MMP-1, MMP-2, MMP-9, and MMP-13 are expressed destructively at elevated levels in the synovial fluid of OA patients.

**A. Collagenases**

The Collagenase group includes three collagenases: collagenase 1 (MMP-1), collagenase 2 (MMP-8), and Collagenase 3 (MMP-13) [23,182,198]. Mesenchymal cells such as fibroblasts and chondrocytes are the main collagenase-producing cells. The cytokines perform predominantly through cell surface receptors and complexes of nuclear oncoproteins mediate the signaling of the cytokines thereby inducing the activation of pro-collagenase gene transcription. Collagen is cleaved in two phases: (i) the first phase is similar to that of interstitial collagenase (ii) the second phase resembles that of gelatinolysis promoted by the fibronectin-like domain [199]. The collagenases cleave fibrillar collagen types I, II, and III at a specific site within the triple helical domain of the collagen molecule, producing characteristic ¾ and ¼ length helical digestion fragments [23,193,182]. In addition to the interstitial fibrillar collagens, these collagenases show specificity for other substrates such as gelatin, casein, aggrecan, laminin, versican, perlecan, fibronectin, and tenascin. Non-ECM substrates of collagenases involve α2 macroglobulin, α1 anti-proteinase inhibitor, α1 antichymotrypsin, insulin-like growth factor binding protein (IGF-BP)-2 and IGF-BP-3, connective tissue growth factor (CTGF) and pro-TGF-β [22,[178], [200],[201], [202]]. Collagenase expression is rate-limiting in the process of fibrillar collagen degradation. Since collagen I, II, and III are very abundant proteins in the human body, the role of collagenases is very important for the maintenance of matrix homeostasis. Collagenases also have a role in skeletal development, bone remodeling, and bone matrix turnover. Collagenases can cleave noncollagenous substrates by the catalytic domain alone but cannot cleave the native fibrillar collagens in the absence of the hemopexin domains. This implies that the expression of their collagenolytic activity needs cooperation between the two domains [[203], [204], [205], [206], [207]].

**(1) Matrix metalloproteinase-1 (MMP-1)**

MMP-1 is considered the prototype for all interstitial collagenases and fibroblast collagenases [22,191,208,209]. It is an enzyme encoded by the MMP-1 gene, the chromosome location of which is 11q22-q23 [21,[181], [210],^201^,^175^,^211^]. MMP-1 is secreted as a protein in response to proinflammatory cytokines (IL-1β and TNFα) and cloned as a cDNA. MMP-1 is secreted as a zymogen where proteolysis removes the N-terminal residues. It is comprised of a prodomain, a catalytic domain, a linker region, and a carboxy-terminal similar to the hemopexin domain. MMP-1 plays an important role in the regulation of cellular behavior taking part in the turnover of collagen fibrils and the cleavage of several non-matrix substrates and cell surface molecules. Type I, II, and II interstitial collagens are broken down by MMP-1 [182,[205], [206], [211]]. Diverse physiological processes including development, tissue morphogenesis, and wound healing are controlled by MMP-1. In healthy conditions, its expression remains low but is stimulated significantly in AC of OA patients [212].

**(2) Matrix metalloproteinase-8 (MMP-8)**

The protease MMP-8, commonly known as neutrophil collagenase, is a protease expressed by both neutrophils and macrophages. Its molecular weight ranges from 50 to 85 kDa upholding varying levels of glycosylation and reflecting the enzyme's latent or activated state [[213], [214], [215], [216], [217], [218]]. Located on chromosome 11q21–q22.3, the MMP8 gene encodes the human MMP-8 protein [21,181,201]. The secondary granules within neutrophils stores the enzyme encoded by this gene and autolytic cleavage activates this enzyme and its activity is regulated by the rate of degranulation. Collagenolytic MMP-8 is released from neutrophils by selective degranulation stimulated by proinflammatory mediators such as IL-1β and TNF-α [213]. MMP-8 is secreted as inactive proproteins which become activated by autolytic cleavage being cleaved by extracellular proteinases. ECM and non-ECM are the substrates for MMP-8 [[219], [220]]. MMP-8 performs as a potential initiator of interstitial collagenolysis at the inflammatory sites.

MMP-8 plays a major role during the inflammatory response and cleaves many substrates including type I, II, and III collagen and cytokines, and enhances ECM breakdown in osteoarthritic cartilage [221,[222], [223]]. In the initial steps of collagen degeneration, the triple helical fibrillar collagen is disrupted by MMP-8 being attacked at a particular site after the Gly-residue of the partial sequences Gly-(lle or Leu) -(Ala-or Leu) which is positioned about three-fourths of the distance down from the N-terminus [20]. Compared to type III, VII, and X fibrillar collagens, type I and II collagens are hydrolyzed more efficiently by MMP-8. Numerous cell types, including neutrophil progenitors and epithelial cells, express it . Its production in arthritic tissue is undoubtedly advantageous because genetic deficiency of MMP-8 worsens arthritis inflammation by inhibiting neutrophil apoptosis and clearance, which leads to an overabundance of neutrophils in the joints [[221], [224], [225], [226], [227], [228]].

**(3) Collagenase-3 (MMP-13)**

**Fig. 5:**
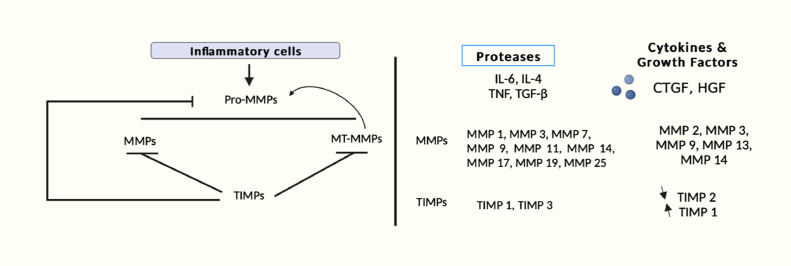
Schematic representation of activation of MMPs during inflammation.

MMP-13 is considered the main catabolic effector in OA and the most studied MMP with respect to its role in cartilage. MMP-13 is a central player in the processes involved in cartilage breakdown. It is the major enzyme that plays a vital role in the cleavage of type II collagen and the irreversible degradation of the AC during OA employing its regulatory factors through the specific signaling pathways [188]. Upgraded MMP-13 activity plays a pivotal role in the induction and pathogenesis of osteoarthritis where AC damage and pathological changes are manifested as synovial hyperplasia and synovitis with diffusion of mononuclear cells in the joints together with cartilage disintegration. The interstitial collagens (I, II, and III) are cleaved by MMP-13 into typical C-terminal and N-terminal polypeptide fragments. During the tissue breakdown the creation of the n-terminus of the major fibromodulin fragment bound to the collagen is not present in normal cartilage [229]. MMP-13 degrades not only type II collagen but also targets other matrix molecules such as proteoglycan, types IV and IX collagen, osteonectin, and perlecan in cartilage [230]. The high expression of MMP-13 in the cartilage of OA patients suggests that the increased expression of MMP-13 is associated with cartilage degradation. Signals induced by stress, inflammation, and differentiation regulate MMP-13 and contribute to joint damage and induction of a phase of the chondrocytes of the articular cartilage to avoid the natural growth and differentiation-boundstate. MMP-13 possesses all the domain characteristics of the MMPs and covers more than 50 % of sequence identity with several collagenase specific residues [231,[227], [230], [232], [233], [234], [235], [236]]. The catalytic zinc ion is connected by histidine residues. MMP-8 shows the greatest effectivity against type I collagen and MMP-13 is five to ten times more active on type II collagen than MMP-1 whose activity is highest on type III collagen [237]. Since the level of expression of MMP-1 is higher than that of MMP-13, a small amount of MMP-1 can overcome the deficiency of degradation capability on type II collagen. The activity of MMP-13 against the fibrillar organization of collagens and its integrity helps in the further cleavage of degradation products resulting in the loss of normal microfibrillar function. MMP-13 can also degrade the aggrecan and perlecan of ECM in the interglobular domain (IGD) and also cleave fibronectin. MMP-1 and MMP-8 are confined to the superficial surface of cartilage but MMP-13 is localized in the deeper layers [238,239]. This fact indicates that synovial cells and neutrophils close to the cartilage mainly produce MMP-1 and MMP-8 whereas MMP-13 is expressed predominantly in chondrocytes [[207], [240], [241], [242]]. MT1-MMP on the cell surface is found to activate MMP-13 and the activation occurs more efficiently in the presence of MMP-2 [173]. In addition to this, proMMP-13 is also activated through the participation of the urokinase-type plasminogen activator-plasmin cascade [243]. The activity of MMP-13 is regulated by the integrated function of these plasmin factors such as endogenous inhibitors, growth factors, MMP-13 transcriptional factors, promoters, receptors, proteases, and hormones. TIMPs and α2-macroglobulin control the activity of the MMP-13 [244]. Due to the wedge shape of the TIMP molecules, they fit into the active site cleft of MMP. TIMPs inhibit MMP-13 in a 1:1 stoichiometric ratio. Activation of MMP-13 is observed to be concentration dependent. MT1-MMP, MT2-MMP, MT3-MMP, and MT5-MMP cannot be inhibited by TIMP-1 whereas the MMP-13 activity is not regulated by TIMP-3 (Fig. 5) [245]. This leads to the progression of OA. In response to enhanced MMP-3 activity possibly through transcriptional regulation and MMP-13 expression, the inhibition of leukocyte release by chondrocytes seems to be elevated in arthritic conditions.

**B. Gelatinases**

The Gelatinases subgroup involves gelatinase A (MMP-2) and gelatinase B (MMP-9) and they are called so due to their ability to degrade gelatine [155,246]. MMP-2 is created constitutively by various types of cells such as macrophages and endothelial cells but MMP-9 is expressed mainly in neutrophils and eosinophils . These enzymes have a distinctive domain termed fibronectin-like domain that contains three repeats of a type II fibronectin motif of nearly 58 amino acids inserted at the catalytic site and are neutral metalloproteinases requiring Ca^2+^ for activity[,]. The fibronectin-like domain is strongly O-glycosylated. Both of the gelatinases are released as inactive pro-forms that are activated extracellularly, similar to all MMPs except for the membrane-bound MT-MMPs [214]. They participate in similar types of proteolytic activity and digest the denatured collagens, gelatins, and various ECM molecules such as type IV, V, and XI collagens, elastin, laminin, vitronectin and aggrecan [187,[247], [248], [249]]. Various non-ECM proteins (chemokines, myelin basic protein, amyloid beta peptide, and substance P) are cleaved by MMP-9 and to a lesser extent by MMP-2 [250].

**(1) MMP-2**

MMP-2 is a Zn^+2^-dependent endopeptidase that is produced as a 72 kDa proenzyme (zymogen form). It is cleaved into a 64 kDa active form and goes through substantial glycosylation. This enzyme is encoded by the MMP-2 gene which is located on chromosome 16q13-q21 at position 12.2 and creates a pro-MMP-2 protein containing 660 amino acids [[176], [192], [251], [252], [253]]. MMP-2 participates in the breakdown of ECM using a variety of substrates. It can break down proteoglycans, fibronectin, elastin, laminin, type I, IV, V, VII, and X collagens [254]. The activity of MMP-2 is important in the repair of damaged tissues, bone remodeling, and inflammation. Unlike the majority of the MMP family, MMP-2 is activated on the cell membrane [27]. With no proteolytic removal of the pro-domain, the activation occurs intracellularly by its S-glutathionylation and extracellularly by proteases [27,192]. Pro-MMP-2 is attracted to the cell surface, and with the aid of a complex of tissue inhibitors of MMP-2 (TIMP-2) and membrane type 1 MMP (MT1-MMP), an autocatalytic cleavage takes place pericellularly [[175], [177], [248], [255]]. TIMP-2 acts as a bridge between pro-MMP-2 and MT-MMP-1 during the activation of MMP-2. The net activity of MT-MMP 1 and MMP-2 is thus dependent upon the concentration of TIMP-2. Cell-cell clustering and clustering of integrins promote the activation of MMP-2. Cell migration is facilitated by MMP-2 through the release of chemoattractants. MMP-2 takes part in both the activation and inhibition of inflammation through the release of proinflammatory mediators like IL-1β and proteolytic degradation of chemoattractants [[256], [257], [258]]. Due to the lack of binding sites in the gene for proinflammatory transcription factors like AP-1 the constitutive and proinflammatory expression of MMP-2 cannot stimulate the degree of the expression. Constitutive expression of MMP-2 and MT1-MMP in arthritic tissues is found possibly due to the lack of a TATA box [259].

**(2) MMP-9**

Gelatinase-B or MMP-9 is a type IV collagenase with a gene that is located on chromosome 20q11.2-q13.1. The synthesis of MMP-9 occurs as a pre-proenzyme consisting of 707 amino acids including 19 amino acid signal peptides. MMP-9 is secreted as an inactive 92-kDa proenzyme and is activated to 83-kDa mature enzyme [[178], [194], [260], [261], [262]]. The pro-MMP-9 is comprised of four domains such as a signal peptide, the amino-terminal propeptide, the zinc-binding catalytic domain with three fibronectin type II repeats, and the carboxy-terminal hemopexin domain [[255], [258], [263],^264^,^188^]. The activation of MMP-9 is mediated through the action of serine proteases, plasmin, or other MMPs such as MMP-3, plasminogen/MMP-3 complex, a complex of pro-MMP-9 and TIMP-1 by the removal of the prodomain and after activation it becomes involved in different physiological processes including the degradation of ECM proteins [178,179]. Articular cartilage loses proteoglycans and collagen by MMP-9 in the final steps of degradation in synovial joint arthritis. MMP-9 activates cytokines and chemokines, truncates IL-8, and stimulates IL-1β and TGF-β to regulate tissue remodeling and the turnover of basement membrane [[180], [181], [182], [183]]. MMP-9 remains stored in neutrophils and is liberated under inflammatory stimulation. An active role is played by MMP-9 in embryonic development, angiogenesis in the growth plate, the immune cell function, the regulation of release of vascular endothelial cell growth factor, fibroblast growth factor-2, and the early period of wound healing [167,175,[184], [185], [186], [189], [190],[250], [265], [266]]. Neutrophils, macrophages, and fibroblasts are found to secrete MMP-9 and it is synthesized in the joint by monocytes and macrophages but minimally by chondrocytes. Osteoclasts, macrophages, trophoblasts, hippocampus neurocytes, and migrating keratinocytes are the only cells that express MMP-9 [178].

Expression of MMP-9 is upregulated in inflammatory OA. MMP-9 is involved in the action of neutrophils in several significant ways, including the degradation of the ECM, stimulation of IL-1, and cleavage of several chemokines [178,175]. Angiogenesis and neovascularization may be significantly influenced by MMP-9. MMP-9 is necessary for the recruitment of endothelial stem cells, a crucial stage in angiogenesis. MMP-9 is controlled by growth factors, chemokines, and other stimulatory signals. MMP-9 has been shown to degrade ECM, initiating and promoting the creation of new blood vessels. Moreover, after being proteolyzed by collagenases, the native type IV, type V, type VII, and type X collagens and elastin, as well as the by-products of type I, type II, and type III collagen, can all be cleaved by this enzyme [196].

Though MMP-2 and MMP-9 have a resemblance in structure and substrate profile, their regulation at the gene transcriptional level differs from each other. The difference in the promoter sections of MMP-2 and MMP-9 genes leads to the difference in the expression nature of the two enzymes. The binding sites for the transcription factor activator protein AP-1 are provided by two TPA-responsive elements (TREs) in the promoter section of the MMP-9 gene [27,267,259,268]. On the other hand, the MMP-2 gene does not possess any binding site for the attachment of inflammatory transcription factors like AP-1 and consequently, MMP-2 transcription is not increased by the proinflammatory stimuli. Along with the prototypic structure of MMPs, MMP-2 and MMP-9 have fibronectin type II-like repeats in the catalytic domain which increases their affinity for gelatin and elastin exceedingly.

**C. Stromelysins**

Stromelysin-1 (MMP-3), stromelysin-2 (MMP-10), and stromelysin-3 (MMP-11) belong to the stromelysin group. These stromelysins possess a similar domain arrangement as the collagenases and do not cleave the triple helical regions of interstitial collagens [173]. Stromelysin is found to play a role in the destruction of connective tissues during disease states. This group of enzymes has broad substrate specificity for the ingredients of the ECM and destructs noncollagenous connective tissue components, including proteoglycans, elastin, fibronectin, laminin, and also some denatured collagens (type II, IV, and IX) [173,[197], [269], [270]]. MMP-3 and MMP-10 have similar structures and substrate specificity and are secreted from the cells as inactive proMMPs. MMP-3 functions as an endogenous proactivators for procollagenase thereby providing additional matrix-degrading capability [271]. MMP-3 cleaves proteins especially at sites where the first three amino acids directly after the cleavage sites are hydrophobic [272].

MMP-3 and MMP-10 differ in their cellular expressions with the expression of MMP-3 in mesenchymal cells and that of MMP-10 in epithelial cells like keratinocytes under specific stimuli. MMP-3 is regulated by growth factors, cytokines, and tumor promoters. MMP-10 regulates the macrophage M_2_ collagenolytic activity without influencing collagen production by fibroblasts [270]. Due to its transcription factor-like action, MMP-3 also seems to be able to increase the synthesis of other MMPs. After being expressed by inflammatory substances, it often manifests in fibroblasts and epithelial cells. The fact that MMP-3 levels are high in osteoarthritic cartilage and the synovium but low in healthy joint tissues may assist in explaining why this enzyme is a potential diagnostic for OA [37,273]. Epidermal growth factor (EGF) is found to enhance stromelysin transcription through the induction of Fos and Jun interacting at the AP-1 site in the promoter. A critical role is also played by platelet-derived growth factors in the induction of stromelysin. Stromelysin transcription is again stimulated by the protein kinase C (PKC) activator, phorbol myristate acetate (PMA) [201]. Similar to other members of the MMP gene family, stromelysin-3 is secreted as an inactive precursor that is stimulated intracellularly by furin to its mature form to express enzymatic activity. In comparison to the extracellular activation of other MMPs, MMP-11 is synthesized in its active form by an intracellular proteolytic incident that takes place within the constitutive secretory pathway. Collagen VI and non-structural ECM component substrates are cleaved by MMP-11 [274]. MMP-11 cannot degrade any important ECM component and shows anti-apoptotic properties by regulating proteinase activity or an inflammatory response. Intracellular activation of MMP-11 is controlled by a 10-amino acid inserted between the pro- and catalytic domains of MMP-11 which is encoded with an Arg-X-Arg-X-Lys-Arg-recognition motif for the proteinase furin. MMP11 is essential for precise collagen VI foldings and for fat tissue cohesion and adipocyte function [155].

**D. Matrilysins**

The Matrilysins group includes MMP-7 (matrilysin-1) and MMP-26 (matrilysin-2) and these MMPs are the smallest among the MMPs [[173], [275]]. The molecular weight of their proenzyme form is of the order 28 kDa whereas that of the active form is ∼19kDa [[156], [173], [197]]. MMP-7 is synthesized by epithelial cells, cardiomyocytes, and macrophages as zinc- and calcium-dependent endopeptidase [202]. The structures of both matrilysins are similar and are comprised of the propeptide and catalytic domains but do not contain the hemopexin domain [173]. The substrate specificity of the matrilysins is the highest among all MMPs. With respect to substrate specificity and the collagenase in the catalytic domain, matrilysins are similar to stromelysin but are devoid of the carboxy-terminal encrypted by other MMP genes [[192], [269], [270], [276]]. The MMP-7 gene is located on chromosome 11q21–q22 [203]. The promoter region of the matrilysin gene contains the promoter elements, AP-1 and PEA3 that respond to the growth factors, oncogenes, and phorbol esters [205]. MMP-7 degrades components of ECM during tissue remodeling including laminin, elastin, tenascin-C, vitronectin, fibronectin, types III, IV, V, IX, X, and XI collagen, type I, II, IV, V gelatins, and proteoglycans [273,203,206]. Besides ECM components it can cleave non-ECM cell surface molecules such as α-defensin, Fas-ligand, β4 integrin, E-cadherin, plasminogen, TNFα [155,277]. MMP-7 can also cleave and activate other MMPs such as MMP-1, −2, and −9 [155]. The specific activity of MMP-7 to the majority of the substrates is highest among the MMPs, even though these substrates overlap with the substrates of other MMPs. MMP-7 plays a critical role in regulating inflammatory reactions and controls biochemical procedures such as activation, degradation, and discharging of non-ECM proteins after their functions [[201], [202], [273], [278]]. Inflammation is promoted by MMP-7 through the discharge of syndecan-1/KC (CXCL8) complexes that permit neutrophil, and transepithelial movement and regulate their influx [273]. Through a mechanism dependent on the urokinase-type plasminogen activator, plasminogen induces macrophages to change promatrilysin into active elastolysin. The matrilysin-deficient macrophages are unable to induce an elastolytic response inspite of the presence of MMP-9 and MMP-12. MMP-26 is secreted as a 28 kDa inactivated enzyme and converted to a 19 kDa active form [209]. MMP-26 contains a prodomain with a cysteine switch sequence PHCGVPDGSD and a catalytic domain with the zinc-binding pattern. It is stored intracellularly in contrast to most other MMPs. Serene proteinase activity is upregulated and the degradation of ECM is increased through the process of inactivation of α_1_-PI by MMP-26 [204]. Due to the fact that MMP-26 can stimulate MMP-9, it is possible that the two enzymes could work in concert as a component of a proteolytic cascade. The principal regulators of the MMP-26 promoter are the T-cell factor-4 (Tcf-4) motif and activator protein-1 (AP-1) [211]. Matrilysins have a broad range of substrate specificity against cell surface molecules, basement membranes, and ECM protein components. The mechanisms of tissue remodeling linked to physiological and pathological processes depend on this extensive degrading activity. The proteolytic digestion of other molecules also seems to be mediated by MAT (e.g.TNF-α precursor, urokinase plasminogen activator). Gelatin, type IV collagen, fibronectin, fibrinogen, and α1 proteinase inhibitor are all degraded by MMP-26. TNF-α and IL-1β stimulate matrilysin, which also has the ability to cleave proteoglycans.

**(c) Membrane-Type MMPs**

Out of the six membrane-type MMPs found, only MT1- and MT3-MMP, have been proven to be implicated in the pathogenesis of OA, with MT1-MMP playing the dominant role [155,156,173]. MT1-MMP contributes to tissue destruction indirectly and directly. Indirectly, MT1-MMP is expressed in both the superficial and transitional zones of OA cartilage where it activates proMMP-2 and proMMP-13 via proteolytic cleavage, allowing these enzymes to degrade collagen, gelatin, and proteoglycans [[213], [242], [279], [280]]. This enzyme has collagenolytic ability and may mediate the resorption of bone [[281], [282]]. The expression pattern of MT3-MMP mimics that of MT1-MMP, suggesting that it may have activities similar to that of MT1-MMP. Thus, the profile of MMPs expressed by connective tissues in arthritic joints is sufficient to destroy the structural collagens that comprise articular cartilage and the adjacent tendons and bones as well as the non-collagen matrix molecules that contribute to joint integrity and function. The expression pattern of MT3-MMP is comparable to that of MT1-MMP, indicating that it may engage in similar functions. Only MT1- and MT3-MMP have been linked to the pathophysiology of OA, with MT1-MMP taking the lead. The remaining five membrane-type MMPs have not been implicated in OA [155,173,[214], [215], [216]].

Membrane-type MMPs (MT-MMPs) consist of two groups: (1) Four type I transmembrane proteins (MMP-14, MMP-15, MMP-16, and MMP-24), (2) two are glycosylphosphatidylinositol (GPI) anchored proteins (MMP-17 and MMP-25). All of them, except for MT4-MMP, can activate proMMP-2 [280,217]. Several ECM components can be destructed by these enzymes, and MT1-MMP possesses collagenolytic activity toward type I, type II and type III collagens. Additionally, MT1-MMP is crucial for angiogenesis. Between β-strand II and III, MT1-MMP (MMP-14), MT2-MMP (MMP-15), MT3-MMP (MMP-16), and MT5-MMP (MMP-24) have an MT loop consisting of an extra 8 residues. For MT1-MMP, this loop is essential for proMMP-2 activation [155].

Adamlysins are membrane-bound MMPs that also break down aggrecan, but more significantly, ADAM-17, a member of the ADAM family, is a TNF-α-converting enzyme (TACE) that stimulates pro-TNF-α. The majority of MMPs are created as latent enzymes that remain dormant. The pro-hormone convertase furin or activator systems like plasminogen activator are usually involved in the conversion to the active enzyme [281].

There are two different forms of MT-MMPs, which contain two glycosylphosphatidylinositol-anchored proteins (MMP-17 and −25) and four type I transmembrane proteins (MMP-14, −15, −16, and −24). At the C-terminus of the propeptide, they all contain a furin-like proprotein convertase recognition sequence RX [R/K]R [173,271,228]. As a result, they are intracellularly activated, and active enzymes are probably expressed on the cell surface. All MT-MMPs can activate proMMP-2, except for MT4-MMP (MMP-17). ProMMP-13 on the cell surface can be activated by MT1-MMP (MMP-14) . On the other hand, MT1-MMP exhibits collagenolytic activity against type I, type II, and type III collagens. The hemopexin-like domain of pro-MMP-2 interacts with the non-inhibitory C-terminal domain of TIMP-2 to form a complex. The activation of pro-MMP-2 by MT1-MMP is required for the formation of this complex [217,283]. To position the pro-MMP-2 pro-domain next to MT1-MMP, the complex first travels to the cell surface where it binds to the active site of MT1-MMP via the free inhibitory N-terminal of TIMP-2. The interactions between the MT1-MMP take place through their hemopexin-like domains, producing a quaternary tetrameric complex with the help of a second MT1-MMP. One MT1-MMP serves as a receptor for the pro-MMP-2-TIMP-2 complex and the other as a pro-MMP-2 activator. By blocking the second MT1-MMP, excess TIMP-2 stops this activation process [26,177].

MT1-MMP (MMP-14), MT2-MMP (MMP-15), MT3-MMP (MMP-16), and MT4-MMP (MMP-17) are the four MT-MMPs that have been discovered in cartilage tissue. It is widely known that chondrocytes and synovial cells express these MT-MMP family members except MMP-14 at low levels. MT1-MMP is found to be the most prevalent in cartilage tissue and is most effectively controlled by cytokines and growth factors [228].

## Signaling pathways

The pathophysiology and progression of OA are mediated by complex regulatory networks with many biochemical signaling pathways, including those of the mitogen-activated protein Kinases (MAPK) [223] and Nuclear factor-kappaB (NF-κB) [284] proteins which control the production of MMP. A cascade of intercellular signals is activated as cytokines bind to receptors on target cells. The inactive protein kinases are activated once the cytokine binds to the receptor located in the cell membrane through phosphorylation . Every cytokine receptor has a connection to one or more JAKs (Janus kinases), which link ligand binding to the tyrosine phosphorylation of different signaling proteins (STATs) recruited to the receptor complex. The Janus kinases, or JAKs, are a unique family of tyrosine kinases that are used by most cytokines to send their signals. The p38MAPK signaling pathway can accelerate the progression of OA by promoting the production of MMPs, which can progressively decrease the synthesis of type II collagen. The p38-MAPK is a class of serine‑threonine protein kinases that are triggered by extracellular stimuli, including inflammatory cytokines, neuron transmitters, cellular stress and adhesion and is found to be correlated with inflammation. The MAPKs pathway is made up of three signal cascade pathways: p38, c-Jun N-terminal kinase (JNK), and extracellular signal-regulated kinase (ERK) 1/2. After dual phosphorylation of tyrosine and threonine residues in a conserved Thr-Xaa–Tyr-motif (where Xaa-is any amino acid) in the activation loop of kinase subdomain VIII, MAPKs become activated. The p38MAPK family comprises four members, namely p38α, p38β, p38γ, and p38δ which have amino acid sequences that are roughly 60 % identical, but they have different expression patterns, substrate specificities, and chemical inhibitor sensitivity. Several signaling pathway-mediated OA processes, including P38MAPK, Wnt, and NF-κB, are linked to IL-1β. Different inhibitors or non-coding RNAs (ncRNAs), block the p38MAPK signaling pathway and suppress the expression of TNF-α and IL-1β, slowing the advancement of OA. MAPKAPK2 (MK2) (a downstream substrate of the p38MAPK) is active in degeneration and mediates the downstream effects of p38 activation on PGE2 release and the production and release of catabolic proteases. p38MAPK suppresses chondrocyte production of MMP-13 in response to catabolic stimulation. For chondrocytes to produce matrix metalloproteinase (MMP) and initiate inflammatory signaling, the signaling molecule p38 mitogen-activated protein kinase is necessary. Catabolic stimulation of human articular chondrocytes activates p38γ, but surprisingly, it decreases MMP-13 synthesis. It is interesting to note that p38MAPK suppression has the potential to reduce chondrocyte inflammation and improve OA, suggesting a potential therapeutic application in OA treatment. NF-κB proteins are a family of transcription factors and are activated by proinflammatory cytokines, chemokines, stress-related substances, and products of extracellular matrix (ECM) degradation. The activation of NF-κB molecules results in the production of several genes that cause articular joint damage and ultimately lead to the onset and progression of OA. The members of the NF-κB/Rel family include c-Rel, NF-κB1 (p50/p105), NF-κB2 (p52/p100), p65 (RelA), and RelB. The most common activated form of NF-κB is a heterodimer made up of (p65) and either the p50 or p52 subunit which is comprised of the transactivation domains required for gene induction. p50 homodimers that bind to a crucial NF-κB–like binding site are the main activators of IL–1–induced collagenase production in synoviocytes. The p50 and p65 heterodimers are closely linked to the activation of inflammatory genes by IL-1 or TNF-α in human monocytes and the antiinflammatory cytokine IL-10 inhibits these effects. The inactive form of NF-κB is found in the cytoplasm and is linked to regulatory proteins known as inhibitors of κB (IκB) (IκBα, IκBβ, and IκBε). As different IκB molecules may regulate different genes in different tissues by inhibiting particular NF-κB subsets, IκB proteins may be appealing targets for particular treatments. There is no scope for detailed discussions of signal pathways of individual cytokines, MMPs and TIMPs.

## Inhibitors

α2-macroglobulin (a plasma glycoprotein) and TIMPs are the two main classes of endogenous inhibitors that control MMP activity. α2-macroglobulin is a proteinase inhibitor that works to prevent proteolytic activity by targeting the proteinase on the α2-macroglobulin "bait" region and the inhibitor undergoes a structural change, which inhibits the enzymatic activity of proteinase [271,[285], [286], [287]]. By trapping the proteinase inside the macroglobulin, it inhibits the majority of proteinases, and the complex is swiftly eliminated by endocytosis, which is mediated by the receptor (low-density lipoprotein receptor-related protein-1). Despite being frequently the first line of defense against pathologic tissue disintegration, this inhibitor cannot penetrate cartilage due to its relatively large size. Therefore, it appears that its function is primarily limited to the inflammatory fluid around the joint.

TIMPs break down the extracellular matrix and remove molecules from cell surfaces.TIMPs are also able to limit ECM deposition, ultimately resulting in a decrease in ECM abundance, by controlling cell function (such as regulation of inflammation, control of the release of TGF-1 activation, or pericyte phenotype) [288,289]. While phenotypes differ between members of the TIMP family, some generalizations can be drawn. In healthy tissues, TIMPs seem to protect the ECM from deterioration, and only the imbalance between MMPs and TIMPs can result in pathological disorders like OA. TIMPs and MMPs respond to inflammation through the proteolytic maturation of cytokines during this process. TIMPs bind non-covalently to the target MMP active site with a 1:1 stoichiometry to inhibit MMP activity. They play a significant role in controlling cellular behavior, tissue remodeling, and ECM turnover [290,291,292]. Different cytokines, chemokines, and growth factors regulate the transcriptional level of the TIMPs, and the expression of the TIMPs can be tissue-specific, constitutive, or inducible [293]. The crystal structures of the TIMP-MMP complexes have been used to clarify the mechanism of TIMP inhibition of MMPs. TIMPs are composed of 184–194 amino acids and consist of two subdomains: N- and C-terminal with ∼125 and 65 amino acids respectively.Three conserved disulfide links are present in each domain, and the N-terminal domain folds as a separate unit with MMP inhibitory action [271,[294], [295],^292^]. The TIMP molecule has a wedge-like overall shape that fits into an MMP's active-site cleft in a similar way to a substrate does. The MMP active site zinc ion is chelated by a conserved cysteine residue at position 1 of the TIMP, which also expels the necessary water molecule [292].The four human TIMPs (TIMP-1, TIMP-2, TIMP-3, and TIMP-4) are broad-spectrum inhibitors of the 23 MMPs found in humans. Four paralogous genes encode TIMPs 1 to 4. Changes in TIMP levels are thought to be significant under pathological situations linked to imbalanced MMP activities because they directly impact the level of MMP activity. TIMP-1 has a comparatively smaller inhibitory range than the other three TIMPs [222]. With its N-terminal-amino group and carbonyl group, Cys1 plays a key role in chelating the active-site zinc and expelling the water molecule linked to the catalytic zinc [290]. Except MT1-MMP, all MMPs examined so far are inhibited by TIMPs. TIMP3 is a major matrix protein that is expressed in a variety of tissues. TIMP-3 differs from the other with respect to inhibitory properties and inhibits aggrecanases, TIMP-3 is not suitable as a therapy option in its natural state due to its lack of selectivity. Though lacking its C-terminal domain, truncated TIMP-3 (N-TIMP-3) is a powerful inhibitor of ADAMTS4 and ADAMTS5, and has been shown to inhibit MMP-1, MMP-2, and (to a lesser extent) MMP-3 [[274], [296]]. By altering the amino acids in the N-terminus of N-TIMP-3, the reactive site has been modified to increase selectivity for ADAMTS4 and ADAMTS5 while minimizing off target effects [297]. TIMP4 is expressed in a relatively small number of tissues, which suggests that it participates in physiological processes unique to those tissues, although little is known about the function of this protein. TIMP-1 also inhibits apoptosis in different cell types. At low to sub-nanomolar doses, TIMP-2 inhibits growth factor-induced cell proliferation. The cell surface molecules are inhibited by TIMP-3. Additionally, TIMP-3 interacts with the angiotensin II receptor, and the overexpression of both TIMP-3 and this receptor together suppresses angiogenesis [298,[228], [243],[284], [285], [286], [287], [288], [289]]. The idea of employing TIMPs as therapeutic agents is interesting because they can inhibit the action of almost all MMPs and are generated by chondrocytes and fibroblasts. The higher levels of MMPs typically outweigh those of TIMPs in the pathologic environment of an arthritic joint, making TIMPs relatively ineffective. This finding has contributed to the growing idea for the clinical application of tailored over-expression of TIMPs [[228], [299]].Since TIMP-3 inhibits MMPs, ADAMTS-4, −5 aggrecanases, and ADAM-17 in OA, it is the top candidate in this respect [300]. However, overexpression of TIMP can have counterintuitive effects, such as enhanced cell proliferation invasion, reduced angiogenesis, and increased apoptosis, in addition to inhibiting the activity of the MMP [167,288]. Despite these drawbacks, it has been suggested that TIMPs could be genetically engineered to be selective for particular MMPs in light of the TIMP/MMP crystal structures. As structural modules, the majority of TIMPs have three things in common: (i) a group that binds to the catalytic zinc; (ii) side chains that bind to various subpockets; and (iii) a peptidomimetic scaffold that mimics the peptide backbone and aligns ZBG and side chains for the best possible interactions with the protein. Although the introduction of weaker zinc-binding groups can reduce efficiency, by enhancing the binding to subpockets, the inhibitory effect can be restored together with higher selectivity. Several highly selective inhibitors (1, 2, 3) were created without the zinc-binding group but with broad, extended hydrophobic substructures since the MMP active region surrounding the Zn2+ ion is generally hydrophobic (such as the S10 pocket) [[291], [292], [293], [297]].

Still now there is no efficient MMP inhibitor for the treatment of arthritic disorders. The design of more selective inhibitors can now be developed through the guidance of the X-ray crystal structures of MMPs. Patients with OA have been reported to have elevated levels of tissue inhibitors of MMPs, but it is unclear whether these levels are due to a compensatory reaction to the enhanced MMPs or if they are accelerating the disease. Treatment methods that stop MMP activity are very appealing, and research has concentrated on inhibitors that are both natural and synthetic that bind to the active site of the enzymes. Although the idea of employing naturally occurring inhibitors is appealing, there are several therapeutic limitations of these inhibitors. Due to the drawbacks of naturally occurring inhibitors, therapeutic compounds that can inhibit MMP activity must be created. These inhibitors were created using the shallow-pocket active sites in collagenases and the deep-pocket active sites in gelatinases [301]. The capacity of a specific medication to specifically block disease-associated MMPs at an early stage may ultimately determine the success of this technique, having the most significant impact on minimizing or preventing irreversible joint degeneration. The stimulation of these signal transduction pathways, which are mediated by inflammatory cytokines, has facilitated the identification of possible therapeutic targets that could control the synthesis of MMPs. MMP gene expression is decreased when inflammatory cytokines are directly blocked to slow arthritis progression .

## Conclusion

1. There are mounting evidence to uphold the critical role of inflammation in the pathogenesis of OA along with the dysregulation within the cytokine networks consisting of inflammatory and anti-inflammatory cytokines. According to the most widely accepted theory to explain the inflammation in OA, the synoviocytes perceive the degraded cartilage fragments coming into contact with the synovium as foreign particles and respond with a protective inflammatory response. Current knowledge about the activity of the key cytokines in the pathogenesis of OA is presented here with the nature and structure of specific receptors. The contribution of cytokines to the enhanced synthesis and release of matrix-decomposing proteolytic enzymes like MMPs and ADAMTS are elaborated emphasizing the potential impact of MMPs on the chondrocytes, synovial cells, articular and periarticular tissues, other cells of the immune system migrating to the site of inflammation.
2. ROS, particularly NO, and oxidative stress perform a crucial role in the angiogenic response to inflammation causing essential chondrocyte death and matrix breakdown and contributing to the start and progress of OA. The signaling molecule NO is thought to be a pro-inflammatory mediator under normal physiological conditions but promotes inflammation when overproduced under abnormal conditions.
3. TIMPs appear to shield the ECM from degradation in healthy tissues, whereas an imbalance between MMPs and TIMPs can lead to pathological conditions like OA. The transcriptional level of the TIMPs is regulated by various cytokines, chemokines, and growth factors, and the expression of the TIMPs can be tissue-specific, constitutive, or inducible. None of the developed synthetic MMP inhibitors has yet completed clinical trials and been commercialized due to the adverse side effects arising from the lack of selectivity and the incomplete understanding of the role of each MMP in the various pathological processes. The following factors have been cited as contributing to MMP inhibitors' subpar performance in clinical trials :(i) Inhibition of other metalloenzymes, (ii) Lack of selectivity within the MMP family, (iii) Inadequate pharmacokinetics, (iv) toxicity, (v) in vivo instability, (vi) low oral availability, and (vii) difficulties to evaluate inhibitory efficacy. A further drawback of MMP inhibitors is their potential to cause side effects on the musculoskeletal system, joint inflammation and pain. The primary arguments put forth were that while some MMPs promote pathology, others have a protective impact. Since there are more than 50 similar metalloproteinases in humans (23 MMPs, 13 ADAMs, and 19 ADAMTSs) and their biological significance is not clearly understood, designing selective metalloproteinase inhibitors presents challenges in identifying the enzymes crucial to disease progression as well as in determining how to screen inhibitors for a specific enzyme or set of enzymes. Comprehensive information on the atomic structure of the specific MMPs has to be targeted as well as the structure of their complexes with inhibitors is a vital requirement.
4. New "second generation" inhibitors, that target a specific MMP with the crystal structures of the various MMPs, may be able to get around some of the above issues. Blocking gene expression is another method for reducing cartilage breakdown caused by MMP. The signal transduction pathways may also be used as potential targets for arthritis therapies. The MMP active site may be more precisely targeted by particular antibodies, which may also reveal regions of the MMP molecule that control the enzyme's extracellular position and substrate specificity. Better selectivity in new MMP inhibitors may increase their specificity, allow them to target particular MMPs in pertinent clinical diseases, and mitigate some of the negative effects. Pharmacological or biological treatments for AC or inflammatory processes are most likely to be ineffective if altered OA joint mechanics are not normalized and native biomechanical pathways are not restored. As numerous pathways and risk factors contribute to mechanical failure of the joint, it would be desirable to identify early OA stages to design more effective, tailored treatments. Personalized OA therapy is the ultimate objective, and current developments in phenotypic classification and tailored medication development may eventually offer a range of effective therapeutic alternatives.

## CRediT authorship contribution statement

**Anwesha Mukherjee:** Formal analysis, Investigation, Methodology, Writing – original draft. **Bodhisatwa Das:** Conceptualization, Funding acquisition, Project administration, Supervision, Writing – review & editing.

## Declaration of competing interest

The authors declare that they have no known competing financial interests or personal relationships that could have appeared to influence the work reported in this paper.

## Data Availability

No data was used for the research described in the article.

## References

[1] Litwic A., Edwards M.H., Dennison E.M., Cooper C. (2013). Epidemiology and burden of osteoarthritis. Br Med Bull.

[2] Hunter D.J., Schofield D., Callander E. (2014). The individual and socioeconomic impact of osteoarthritis. Nat Rev Rheumatol.

[3] Sarzi-Puttini P., Cimmino M.A., Scarpa R., Caporali R., Parazzini F., Zaninelli A., Atzeni F., Canesi B. (2005). Osteoarthritis: an overview of the disease and its treatment strategies. Semin Arthritis Rheum.

[4] Bhosale A.M., Richardson J.B. (2008). Articular cartilage: structure, injuries and review of management. Br Med Bull.

[5] Sophia Fox A.J., Bedi A., Rodeo S.A. (2009). The basic science of articular cartilage: structure, composition, and function. Sports Health.

[6] Buckwalter J.A., Mankin H.J., Grodzinsky A.J. (2005). Instructional course lectures-american academy of orthopaedic surgeons.

[7] Ng H.Y., Lee K.-X.A., Shen Y.-F. (2017). Articular cartilage: structure, composition, injuries and repair. JSM Bone Joint Dis.

[8] Howell D.S. (1980). Biology of Cartilage Cells. R. A. Stockwell. Cambridge, Cambridge University Press, 1979. 329 pages; illustrated. Arthr Rheumat.

[9] Buckwalter J., Mankin H. (1998). Articular cartilage: tissue design and chondrocyte-matrix interactions. Instr Course Lect.

[10] N.P. Cohen, Composition and dynamics of articular cartilage: structure, function, and maintaining ∼ e a l t h yState, (1998). 10.2519/jospt.1998.28.4.203 9785256

[11] Mansour J.M. (2003). Biomechanics of cartilage. Kinesiol: Mech Pathomech Hum Movement.

[12] Poole C.A. (1997). Articular cartilage chondrons: form, function and failure. J Anatomy.

[13] Chow Y.Y., Chin K.-Y. (2020). The role of inflammation in the pathogenesis of osteoarthritis. Mediat Inflamm..

[14] P. Brooks, Inflammation as an important feature of osteoarthritis, (n.d.). PMC2572543 14710513

[15] Benito M.J. (2005). Synovial tissue inflammation in early and late osteoarthritis. Ann Rheum Dis.

[16] Wojdasiewicz P., Poniatowski Ł.A., Szukiewicz D. (2014). The role of inflammatory and anti-inflammatory cytokines in the pathogenesis of osteoarthritis. Mediat Inflamm..

[17] Fábio dos Santos Duarte Lana J., Rodrigues B.L., Toumi H., Mazor M. (2019). Osteoarthritis biomarkers and treatments.

[18] Woodell-May J.E., Sommerfeld S.D. (2020). Role of inflammation and the immune system in the progression of osteoarthritis. J Orthop Res.

[19] Goldring S.R., Goldring M.B. (2004). The role of cytokines in cartilage matrix degeneration in osteoarthritis. Clin Orthopaed Relat Res.

[20] Goldring M.B. (2000). Osteoarthritis and cartilage: the role of cytokines. Curr Rheumatol Rep.

[21] Robinson W., Lepus C., Wang Q., Raghu H., Mao R., Lindstrom T., Sokolove J. (2016). Low-grade inflammation as a key mediator of the pathogenesis of osteoarthritis. Nat Rev Rheumatol.

[22] Mehana E.-S.E., Khafaga A.F., El-Blehi S.S. (2019). The role of matrix metalloproteinases in osteoarthritis pathogenesis: an updated review. Life Sci.

[23] Laronha H., Caldeira J. (2020). Structure and function of human matrix metalloproteinases. Cells.

[24] Loffek S., Schilling O., Franzke C.-W. (2011). Biological role of matrix metalloproteinases: a critical balance. Eur Respirat J.

[25] Burrage P., Mix K., Brinckerhoff C. (2006). Matrix metalloproteinases: role in arthritis. Front Biosci: A J Virtual Lib.

[26] Visse R., Nagase H. (2003). Matrix metalloproteinases and tissue inhibitors of metalloproteinases: structure, function, and biochemistry. Circ Res.

[27] Hannoodee S., Nasuruddin D.N. (2021). StatPearls [Internet].

[28] Ryan G.B., Majno G. (1977). Acute inflammation. A review. Am J Pathol.

[29] Kumar R., Clermont G., Vodovotz Y., Chow C.C. (2004). The dynamics of acute inflammation. J Theor Biol.

[30] Scanzello C.R. (2017). Role of low-grade inflammation in osteoarthritis. Curr Opin Rheumatol.

[31] Chen L., Deng H., Cui H., Fang J., Zuo Z., Deng J., Li Y., Wang X., Zhao L. (2018). Inflammatory responses and inflammation-associated diseases in organs. Oncotarget.

[32] Sokolove J., Lepus C.M. (2013). Role of inflammation in the pathogenesis of osteoarthritis: latest findings and interpretations. Ther Adv Musculoskelet Dis.

[33] Bonnet C.S. (2005). Osteoarthritis, angiogenesis and inflammation. Rheumatology.

[34] Goldring M.B., Otero M. (2011). Inflammation in osteoarthritis. Curr Opin Rheumatol.

[35] Liu S., Deng Z., Chen K., Jian S., Zhou F., Yang Y., Fu Z., Xie H., Xiong J., Zhu W. (2022). Cartilage tissue engineering: from proinflammatory and anti‑inflammatory cytokines to osteoarthritis treatments (Review). Mol Med Rep.

[36] Fox A.J.S., Bedi A., Rodeo S.A. (2012). The basic science of human knee menisci: structure, composition, and function. Sports Health.

[37] MacConaill M., Barnett C., Davies D. (1961).

[38] Archer C.W., Dowthwaite G.P., Francis-West P. (2003). Development of synovial joints. Birth Defect Res C.

[39] Barland P., Novikoff A.B., Hamerman D. (1962). Electron microscopy of the human synovial membrane. J Cell Biol.

[40] Scanzello C.R., Goldring S.R. (2012). The role of synovitis in osteoarthritis pathogenesis. Bone.

[41] Ishii H., Tanaka H., Katoh K., Nakamura H., Nagashima M., Yoshino S. (2002). Characterization of infiltrating T cells and Th1/Th2-type cytokines in the synovium of patients with osteoarthritis. Osteoarthr Cartilage.

[42] J. Bondeson, A.B. Blom, S. Wainwright, C. Hughes, B. Caterson, The role of synovial macrophages and macrophage- produced mediators in driving inflammatory and destructive responses in osteoarthritis, (n.d.). 10.1002/art.27290 20187160

[43] Mathiessen A., Conaghan P.G. (2017). Synovitis in osteoarthritis: current understanding with therapeutic implications. Arthritis Res Ther.

[44] Bondeson J., Blom A.B., Wainwright S., Hughes C., Caterson B., Van Den Berg W.B. (2010). The role of synovial macrophages and macrophage-produced mediators in driving inflammatory and destructive responses in osteoarthritis. Arthr Rheumat-Arthr Care Res.

[45] Buckwalter J., Martin J., Mankin H. (2000). Synovial joint degeneration and the syndrome of osteoarthritis. Instr Course Lect.

[46] D.E. Nanus, A. Badoume, S.N. Wijesinghe, A.M. Halsey, P. Hurley, Z. Ahmed, R. Botchu, E.T. Davis, M.A. Lindsay, S.W. Jones, Synovial tissue from sites of joint pain in knee osteoarthritis patients exhibits a differential phenotype with distinct fibroblast subsets, EBioMedicine. 72 (2021) 103618. 10.1016/j.ebiom.2021.103618. PMC8511845 34628351

[47] Lee J.M., Fu F.H. (2000). The meniscus: basic science and clinical applications. Oper Tech Orthop.

[48] Vadodaria K., Kulkarni A., Santhini E., Vasudevan P. (2019). Materials and structures used in meniscus repair and regeneration: a review. Biomedicine (Taipei).

[49] Hedbom E., Häuselmann H.J. (2002). Molecular aspects of pathogenesis in osteoarthritis: the role of inflammation. Cell Mol Life Sci (CMLS).

[50] Molnar V., Matišić V., Kodvanj I., Bjelica R., Jeleč Ž., Hudetz D., Rod E., Čukelj F., Vrdoljak T., Vidović D., Starešinić M., Sabalić S., Dobričić B., Petrović T., Antičević D., Borić I., Košir R., Zmrzljak U.P., Primorac D. (2021). Cytokines and chemokines involved in osteoarthritis pathogenesis. IJMS.

[51] Vangsness C.T., Burke W., Narvy S., MacPhee R., Fedenko A. (2011). Human knee synovial fluid cytokines correlated with grade of knee osteoarthritis: a pilot study. Bull NYU Hosp Jt Dis.

[52] Melo-Florián A. (2011). IL-1 and its role in osteoarthritis. Open J Med.

[53] Lopez-Castejon G., Brough D. (2011). Understanding the mechanism of IL-1β secretion. Cytokine Growth Factor Rev.

[54] Dinarello C.A. (2013).

[55] Piccioli P., Rubartelli A. (2013).

[56] Roman-Blas J.A., Jimenez S.A. (2006). NF-κB as a potential therapeutic target in osteoarthritis and rheumatoid arthritis. Osteoarthr Cartilage.

[57] Boraschi D., Tagliabue A. (2013).

[58] Tak P.P., Firestein G.S. (2001). NF-κB: a key role in inflammatory diseases. J Clin Investig.

[59] Caron J.P., Fernandes J.C., Martel-Pelletier J., Tardif G., Mineau F., Geng C., Pelletier J. (1996). Chondroprotective effect of intraarticular injections of interleukin-1 receptor antagonist in experimental osteoarthritis. Suppression of collagenase-1 expression. Arthr Rheumat: Off J Am Coll Rheumatol.

[60] Palmer G., Guerne P.-A., Mezin F., Maret M., Guicheux J., Goldring M.B., Gabay C. (2002). Production of interleukin-1 receptor antagonist by human articular chondrocytes. Arthritis Res Ther.

[61] M.B. Sadouk, J.P. Pelletier, G. Tardif, K. Kiansa, J.M. Cloutier, J. Martel-Pelletier, Human synovial fibroblasts coexpress IL-1 receptor type I and type II mRNA. The increased level of the IL-1 receptor in osteoarthritic cells is related to an increased level of the type I receptor, (n.d.). 7564267

[62] Martel-Pelletier J., Mccollum R., Dibattista J., Faure M.-P., Chin J.A., Fournier S., Sarfati M., Pelletier J.-P. (1992). The interleukin-1 receptor in normal and osteoarthritic human articular chondrocytes. Identification as the type I receptor and analysis of binding kinetics and biologic function: the interleukin-1 receptor in normal and osteoarthritic human articular chondrocytes. Identification as the type I receptor and analysis of binding ki. Arthr Rheumat.

[63] Martin M.U., Wesche H. (2002). Summary and comparison of the signaling mechanisms of the Toll/interleukin-1 receptor family. Biochim Biophys Acta (BBA) - Mol Cell Res.

[64] Verma P., Dalal K. (2011). ADAMTS-4 and ADAMTS-5: key enzymes in osteoarthritis. J. Cell. Biochem.

[65] Afonso V., Champy R., Mitrovic D., Collin P., Lomri A. (2007). Reactive oxygen species and superoxide dismutases: role in joint diseases. Joint Bone Spine.

[66] El Mansouri F.E., Chabane N., Zayed N., Kapoor M., Benderdour M., Martel-Pelletier J., Pelletier J.-P., Duval N., Fahmi H. (2011). Contribution of H3K4 methylation by SET-1A to interleukin-1-induced cyclooxygenase 2 and inducible nitric oxide synthase expression in human osteoarthritis chondrocytes. Arthr Rheumat.

[67] Gilman S.C., Chang J., Zeigler P.R., Uhl J., Mochan E. (1988). Interleukin-1 activates phospholipase a2 in human synovial cells. Arthr Rheumat.

[68] Hardy M.M., Seibert K., Manning P.T., Currie M.G., Woerner B.M., Edwards D., Koki A., Tripp C.S. (2002). Cyclooxygenase 2-dependent prostaglandin E2 modulates cartilage proteoglycan degradation in human osteoarthritis explants. Arthr Rheumat.

[69] Kumar A., Arshad M., Singh A., Khan H., Swaroop S. (2018). Association of cytokine TNF-α in development of osteoarthritis: a comprehensive study. J Ecophysiol Occup Health.

[70] Bodmer J.-L., Schneider P., Tschopp J. (2002). The molecular architecture of the TNF superfamily. Trends Biochem Sci.

[71] Grell M., Douni E., Wajant H., Löhden M., Clauss M., Maxeiner B., Georgopoulos S., Lesslauer W., Kollias G., Pfizenmaier K., Scheurich P. (1995). The transmembrane form of tumor necrosis factor is the prime activating ligand of the 80kDa tumor necrosis factor receptor. Cell.

[72] MacEwan D.J. (2002). TNF receptor subtype signalling: differences and cellular consequences. Cell. Signal.

[73] Alaaeddine N., DiBattista J., Pelletier J., Cloutier J., Kiansa K., Dupuis M., Martel-Pelletier J. (1997). Osteoarthritic synovial fibroblasts possess an increased level of tumor necrosis factor-receptor 55 (TNF-R55) that mediates biological activation by TNF-alpha. J Rheumatol.

[74] Steenvoorden M., Bank R., Ronday H., Toes R., Huizinga T., DeGroot J. (2007). Fibroblast-like synoviocyte-chondrocyte interaction in cartilage degradation. Clin Exp Rheumatol.

[75] Oregón-Romero E., Vázquez-Del Mercado M., Navarro-Hernández R.E., Torres-Carrillo N., Martínez-Bonilla G., Estrada-García I., Rangel-Villalobos H., Muñoz-Valle J.F. (2006). Tumor necrosis factor receptor 2 M196R polymorphism in rheumatoid arthritis and osteoarthritis: relationship with sTNFR2 levels and clinical features. Rheumatol Int.

[76] Guo F., Lai Y., Tian Q., Lin E.A., Kong L., Liu C. (2010). Granulin-epithelin precursor (GEP) binds directly to ADAMTS-7 and ADAMTS-12 and inhibits their degradation of cartilage oligomeric matrix protein. Arthr Rheumat.

[77] Jian J., Konopka J., Liu C. (2013). Insights into the role of progranulin in immunity, infection, and inflammation. J Leukoc Biol.

[78] Perera L.P. (2001). Interleukin 15: its role in inflammation and immunity. Inflammation.

[79] Steel J.C., Waldmann T.A., Morris J.C. (2012). Interleukin-15 biology and its therapeutic implications in cancer. Trends Pharmacol Sci.

[80] Waldmann T.A., Tagaya Y. (1999). The multifaceted regulation of interleukin-15 expression and the role of this cytokine in nk cell differentiation and host response to intracellular pathogens. Annu Rev Immunol.

[81] Sun J.-M., Sun L.-Z., Liu J., Su B., Shi L. (2013). Serum interleukin-15 levels are associated with severity of pain in patients with knee osteoarthritis. Dis Mark.

[82] Scanzello C.R., Umoh E., Pessler F., Diaz-Torne C., Miles T., DiCarlo E., Potter H.G., Mandl L., Marx R., Rodeo S., Goldring S.R., Crow M.K. (2009). Local cytokine profiles in knee osteoarthritis: elevated synovial fluid interleukin-15 differentiates early from end-stage disease. Osteoarthritis Cartilage.

[83] Olsen S.K., Ota N., Kishishita S., Kukimoto-Niino M., Murayama K., Uchiyama H., Toyama M., Terada T., Shirouzu M., Kanagawa O., Yokoyama S. (2007). Crystal structure of the interleukin-15·interleukin-15 receptor α complex. J Biol Chem.

[84] Jakobisiak M., Golab J., Lasek W. (2011). Interleukin 15 as a promising candidate for tumor immunotherapy. Cytokine Growth Factor Rev.

[85] Zhang X., Angkasekwinai P., Dong C., Tang H. (2011). Structure and function of interleukin-17 family cytokines. Prot Cell.

[86] Kolls J.K., Lindén A. (2004). Interleukin-17 Family Members and Inflammation. Immunity.

[87] Chang S.H., Dong C. (2011). Signaling of interleukin-17 family cytokines in immunity and inflammation. Cell Signal.

[88] Pawłowska J., Mikosik A., Soroczynska-Cybula M., Jóźwik A., Łuczkiewicz P., Mazurkiewicz S., Lorczyński A., Witkowski J.M., Bryl E. (2010). Different distribution of CD4 and CD8 T cells in synovial membrane and peripheral blood of rheumatoid arthritis and osteoarthritis patients. Folia Histochem Cytobiol.

[89] Honorati M.C., Bovara M., Cattini L., Piacentini A., Facchini A. (2002). Contribution of interleukin 17 to human cartilage degradation and synovial inflammation in osteoarthritis. Osteoarthr Cartilage.

[90] Attur M.G., Patel R.N., Abramson S.B., Amin A.R. (1997). Interleukin-17 up-regulation of nitric oxide production in human osteoarthritis cartilage. Arthr Rheumat: Off J Am Coll Rheumatol.

[91] LeGrand A., Fermor B., Fink C., Pisetsky D.S., Weinberg J.B., Vail T.P., Guilak F. (2001). Interleukin-1, tumor necrosis factor?, and interleukin-17 synergistically up-regulate nitric oxide and prostaglandin E2 production in explants of human osteoarthritic knee menisci. Arthr Rheumat.

[92] Zenobia C., Hajishengallis G. (2015). Basic biology and role of interleukin-17 in immunity and inflammation. Periodontol 2000.

[93] Mimpen J.Y., Baldwin M.J., Cribbs A.P., Philpott M., Carr A.J., Dakin S.G., Snelling S.J.B. (2021). Interleukin-17A causes osteoarthritis-like transcriptional changes in human osteoarthritis-derived chondrocytes and synovial fibroblasts in vitro. Front Immunol.

[94] Ghayur T., Banerjee S., Hugunin M., Butler D., Herzog L., Carter A., Quintal L., Sekut L., Talanian R., Paskind M., Wong W., Kamen R., Tracey D., Alien H. (1997). Caspase-1 processes IFN-γ-inducing factor and regulates LPS-induced IFN- γ production. Nature.

[95] Korn T., Bettelli E., Oukka M., Kuchroo V.K. (2009). IL-17 and Th17 cells. Annu Rev Immunol.

[96] Okamura H., Tsutsui H., Komatsu T., Yutsudo M., Hakura A., Tanimoto T., Torigoe K., Okura T., Nukada Y., Hattori K., Akita K., Namba M., Tanabe F., Konishi K., Fukuda S., Kurimoto M. (1995). Cloning of a new cytokine that induces IFN-γ production by T cells. Nature.

[97] Denoble A.E., Huffman K.M., Stabler T.V., Kelly S.J., Hershfield M.S., McDaniel G.E., Coleman R.E., Kraus V.B. (2011). Uric acid is a danger signal of increasing risk for osteoarthritis through inflammasome activation. Proc Natl Acad Sci USA.

[98] Olee T., Hashimoto S., Quach J., Lotz M. (1999). IL-18 is produced by articular chondrocytes and induces proinflammatory and catabolic responses1. J Immunol.

[99] Udagawa N., Horwood N.J., Elliott J., Mackay A., Owens J., Okamura H., Kurimoto M., Chambers T.J., Martin T.J., Gillespie M.T. (1997). Interleukin-18 (interferon-γ–inducing factor) is produced by osteoblasts and acts via granulocyte/macrophage colony-stimulating factor and not via interferon-γ to inhibit osteoclast formation. J Exp Med.

[100] Peng C., Cao J., Xiao T., Peng C., Yang H., Chen X., Fang J. (2006). [Concentration of IL-18 and PGE2 in synovial fluid in patients with osteoarthritis and its significance]. Zhong Nan Da Xue Xue Bao Yi Xue Ban.

[101] Saha N., Moldovan F., Tardif G., Pelletier J.-P., Cloutier J.-M., Martel-Pelletier J. (1999). Interleukin-1?-converting enzyme/caspase-1 in human osteoarthritic tissues: localization and role in the maturation of interleukin-1? and interleukin-18. Arthritis Rheumat.

[102] Möller B., Paulukat J., Nold M., Behrens M., Kukoc-Zivojnov N., Kaltwasser J.P., Pfeilschifter J., Mühl H. (2003). Interferon-γ induces expression of interleukin-18 binding protein in fibroblast-like synoviocytes. Rheumatology.

[103] Born T.L., Thomassen E., Bird T.A., Sims J.E. (1998). Cloning of a novel receptor subunit, AcPL, required for interleukin-18 signaling. J Biol Chem.

[104] Torigoe K., Ushio S., Okura T., Kobayashi S., Taniai M., Kunikata T., Murakami T., Sanou O., Kojima H., Fujii M., Ohta T., Ikeda M., Ikegami H., Kurimoto M. (1997). Purification and characterization of the human interleukin-18 receptor. J Biol Chem.

[105] Dai S.-M. (2005). Implication of interleukin 18 in production of matrix metalloproteinases in articular chondrocytes in arthritis: direct effect on chondrocytes may not be pivotal. Ann Rheum Dis.

[106] Futani H., Okayama A., Matsui K., Kashiwamura S., Sasaki T., Hada T., Nakanishi K., Tateishi H., Maruo S., Okamura H. (2002). Relation between interleukin-18 and PGE2 in synovial fluid of osteoarthritis: a potential therapeutic target of cartilage degradation. J Immunother.

[107] Li Y., Jiang J., Yang D., Wang F., Mao Z. (2009). [Determination of the concentrations of interleukin-18 and other cytokines in the synovial fluid in patients with osteoarthritis]. Nan Fang Yi Ke Da Xue Xue Bao.

[108] Wang F., Jiang J., Wang F., Fu Z., Zhang Z. (2010). [Expressions of interleukin 18 and prostaglandin E2 and their correlation in the synoviocytes of patients with osteoarthritis]. Nan Fang Yi Ke Da Xue Xue Bao.

[109] Wang Y., Xu D., Long L., Deng X., Tao R., Huang G. (2014). Correlation between plasma, synovial fluid and articular cartilage Interleukin-18 with radiographic severity in 33 patients with osteoarthritis of the knee. Clin Exp Med.

[110] Cho M.-L., Jung Y.O., Moon Y.-M., Min S.-Y., Yoon C.-H., Lee S.-H., Park S.-H., Cho C.-S., Jue D.-M., Kim H.-Y. (2006). Interleukin-18 induces the production of vascular endothelial growth factor (VEGF) in rheumatoid arthritis synovial fibroblasts via AP-1-dependent pathways. Immunol Lett.

[111] Wagner S., Fritz P., Einsele H., Sell S., Saal J.G. (1997). Evaluation of synovial cytokine patterns in rheumatoid arthritis and osteoarthritis by quantitative reverse transcription polymerase chain reaction. Rheumatol Int.

[112] Powers R., Garrett D.S., March C.J., Frieden E.A., Gronenborn A.M., Clore G.M. (1993). The high-resolution, three-dimensional solution structure of human interleukin-4 determined by multidimensional heteronuclear magnetic resonance spectroscopy. Biochemistry.

[113] Nelms K., Keegan A.D., Zamorano J., Ryan J.J., Paul W.E. (1999). THE IL-4 RECEPTOR: signaling mechanisms and biologic functions. Annu Rev Immunol.

[114] LaPorte S.L., Juo Z.S., Vaclavikova J., Colf L.A., Qi X., Heller N.M., Keegan A.D., Garcia K.C. (2008). Molecular and structural basis of cytokine receptor pleiotropy in the interleukin-4/13 system. Cell.

[115] Silvestri T., Pulsatelli L., Dolzani P., Facchini A., Meliconi R. (2006). Elevated serum levels of soluble interleukin-4 receptor in osteoarthritis. Osteoarthr Cartilage.

[116] Yorimitsu M., Nishida K., Shimizu A., Doi H., Miyazawa S., Komiyama T., Nasu Y., Yoshida A., Watanabe S., Ozaki T. (2008). Intra-articular injection of interleukin-4 decreases nitric oxide production by chondrocytes and ameliorates subsequent destruction of cartilage in instability-induced osteoarthritis in rat knee joints. Osteoarthritis Cartilage.

[117] van Meegeren M.E.R., Roosendaal G., Jansen N.W.D., Wenting M.J.G., van Wesel A.C.W., van Roon J.A.G., Lafeber F.P.J.G. (2012). IL-4 alone and in combination with IL-10 protects against blood-induced cartilage damage. Osteoarthritis Cartilage.

[118] Mueller T.D., Zhang J.-L., Sebald W., Duschl A. (2002). Structure, binding, and antagonists in the IL-4/IL-13 receptor system. Biochim Biophys Acta (BBA) - Mol Cell Res.

[119] Demazière A., Leek R., Athanasou N. (1992). Histological distribution of the interleukin-4 receptor (IL4R) within the normal and pathological synovium. Rev Rhum Mal Osteoartic.

[120] Alaaeddine N., Di Battista J.A., Pelletier J.-P., Kiansa K., Cloutier J.-M., Martel-Pelletier J. (1999). Inhibition of tumor necrosis factor?-induced prostaglandin E2 production by the antiinflammatory cytokines interleukin-4, interleukin-10, and interleukin-13 in osteoarthritic synovial fibroblasts: distinct targeting in the signaling pathways. Arthrit Rheumatism.

[121] Wlodaver A., Pavlovsky A., Gustchina A. (1992). Crystal structure of human recombinant interleukin-4 at 2.25Å resolution. FEBS Lett.

[122] Vieira P., de Waal-Malefyt R., Dang M.N., Johnson K.E., Kastelein R., Fiorentino D.F., deVries J.E., Roncarolo M.G., Mosmann T.R., Moore K.W. (1991). Isolation and expression of human cytokine synthesis inhibitory factor cDNA clones: homology to Epstein-Barr virus open reading frame BCRFI. Proc Natl Acad Sci USA.

[123] Nishida K., Yorimitsu M., Komiyama T., Kadota Y., Tetsunaga T., Yoshida A., Kubota S., Takigawa M., Ozaki T. (2008). Interleukin-4 downregulates the cyclic tensile stress-induced matrix metalloproteinases-13 and cathepsin B expression by rat normal chondrocytes. Acta Med Okayama.

[124] Zurawski G., de Vries J.E. (1994). Interleukin 13, an interleukin 4-like cytokine that acts on monocytes and B cells, but not on T cells. Immunol Today.

[125] Tetlow L.C., Adlam D.J., Woolley D.E. (2001). Matrix metalloproteinase and proinflammatory cytokine production by chondrocytes of human osteoarthritic cartilage: associations with degenerative changes. Arthrit Rheumat.

[126] Tudorachi N.B., Totu E.E., Fifere A., Ardeleanu V., Mocanu V., Mircea C., Isildak I., Smilkov K., Cărăuşu E.M. (2021). The implication of reactive oxygen species and antioxidants in knee osteoarthritis. Antioxidants.

[127] Zdanov A., Schalk-Hihi C., Gustchina A., Tsang M., Weatherbee J., Wlodawer A. (1995). Crystal structure of interleukin-10 reveals the functional dimer with an unexpected topological similarity to interferon γ. Structure.

[128] Tan J.C., Indelicato S.R., Narula S.K., Zavodny P.J., Chou C.C. (1993). Characterization of interleukin-10 receptors on human and mouse cells. J Biol Chem.

[129] Kotenko S.V., Krause C.D., Izotova L.S., Pollack B.P., Wu W., Pestka S. (1997). Identification and functional characterization of a second chain of the interleukin-10 receptor complex. EMBO J.

[130] Liu Y., Wei S.H., Ho A.S., de Waal Malefyt R., Moore K.W. (1994). Expression cloning and characterization of a human IL-10 receptor. J Immunol.

[131] Eisenmesser E.Z., Horita D.A., Altieri A.S., Byrd R.A., Wright P.E. (2001). Solution structure of interleukin-13 and insights into receptor engagement11Edited by. J Mol Biol.

[132] A. Minty, P. Chalon, J.-M. Derocq, X. Dumont, J.-C. Guillemot, M. Kaghad, C. Labit, P. Leplatois, P. Liauzun, B. Miloux, C. Minty, P. Casellas, G. Loison, J. Lupker, D. Shire, P. Ferrara, D. Caput, lnterleukin-13 is a new human lymphokine regulating inflammatory and immune responses, Nature. 362 (1993) 248–50. 10.1038/362248a0. 8096327

[133] Iwaszko M., Biały S., Bogunia-Kubik K. (2021). Significance of Interleukin (IL)-4 and IL-13 in Inflammatory arthritis. Cells.

[134] Eisenmesser E.Z., Horita D.A., Byrd R.A. (2001). Letter to the Editor: secondary structure and backbone resonance assignments for human interleukin-13. J Biomol NMR.

[135] Rael E.L., Lockey R.F. (2011). Interleukin-13 Signaling and Its Role in Asthma. World Allergy Org J.

[136] Lupardus P.J., Birnbaum M.E., Garcia K.C. (2010). Molecular basis for shared cytokine recognition revealed in the structure of an unusually high affinity complex between IL-13 and IL-13Rα2. Structure.

[137] Nathan C., Xie Q. (1994). Nitric oxide synthases: roles, tolls, and controls. Cell.

[138] Coleman J.W. (2001). Nitric oxide in immunity and inflammation. Int Immunopharmacol.

[139] Fermor B., Christensen S., Youn I., Cernanec J., Davies C., Weinberg J. (2007). Oxygen, nitric oxide and articular cartilage. Eur Cell Mater.

[140] Abramson S.B. (2008). Osteoarthritis and nitric oxide. Osteoarthritis Cartilage.

[141] Abramson S.B., Amin A.R., Clancy R.M., Attur M. (2001). The role of nitric oxide in tissue destruction. Best Pract Res Clin Rheumatol.

[142] Hess D.T., Matsumoto A., Kim S.-O., Marshall H.E., Stamler J.S. (2005). Protein S-nitrosylation: purview and parameters. Nat Rev Mol Cell Biol.

[143] Ahmad N., Ansari M.Y., Haqqi T.M. (2020). Role of iNOS in osteoarthritis: pathological and therapeutic aspects. J Cell Physiol.

[144] Abramson S.B. (2008). Nitric oxide in inflammation and pain associated with osteoarthritis. Arthritis Res Ther.

[145] Jang D., Murrell G.A.C. (1998). Nitric Oxide in Arthritis. Free Radic Biol Med.

[146] Kolios G., Valatas V., Ward S.G. (2004). Nitric oxide in inflammatory bowel disease: a universal messenger in an unsolved puzzle. Immunology.

[147] Murphy G., Knäuper V., Atkinson S., Butler G., English W., Hutton M., Stracke J., Clark I. (2002). Matrix metalloproteinases in arthritic disease. Arthritis Res Ther.

[148] Rose B.J., Kooyman D.L. (2016). A tale of two joints: the role of matrix metalloproteases in cartilage biology. Dis Mark.

[149] Woessner J.F., Nagase H. (2000).

[150] Scher J.U., Pillinger M.H., Abramson S.B. (2007). Nitric oxide synthases and osteoarthritis. Curr Rheumatol Rep.

[151] Van Lint P., Libert C. (2007). Chemokine and cytokine processing by matrix metalloproteinases and its effect on leukocyte migration and inflammation. J Leukoc Biol.

[152] Parks W.C., Wilson C.L., López-Boado Y.S. (2004). Matrix metalloproteinases as modulators of inflammation and innate immunity. Nat Rev Immunol.

[153] Chakraborti S., Mandal M., Das S., Mandal A., Chakraborti T. (2003). Regulation of matrix metalloproteinases: an overview. Mol Cell Biochem.

[154] Klein T., Bischoff R. (2011). Physiology and pathophysiology of matrix metalloproteases. Amino Acids.

[155] A.V. Chernov, A.Y. Strongin, Epigenetic regulation of matrix metalloproteinases and their collagen substrates in cancer, 2 (2011) 135–47. 10.1515/bmc.2011.017. PMC3138141 21779312

[156] Amar S., Smith L., Fields G.B. (2017). Matrix metalloproteinase collagenolysis in health and disease. Biochim Biophys Acta (BBA) - Mol Cell Res.

[157] Young D.A., Barter M.J., Wilkinson D.J. (2019). Recent advances in understanding the regulation of metalloproteinases. F1000Res.

[158] Nagase H., Woessner J.F. (1999). Matrix metalloproteinases. J Biol Chem.

[159] Verma R.P., Hansch C. (2007). Matrix metalloproteinases (MMPs): chemical–biological functions and (Q)SARs. Bioorg Med Chem.

[160] Goodwin L. (2019).

[161] Cui N., Hu M., Khalil R.A., Khalil R.A. (2017). Progress in molecular biology and translational science.

[162] Murphy G., Nagase H. (2008). Progress in matrix metalloproteinase research. Mol Asp Med.

[163] Sternlicht M.D., Werb Z. (2001). How matrix metalloproteinases regulate cell behavior. Annu Rev Cell Dev Biol.

[164] Page-McCaw A., Ewald A.J., Werb Z. (2007). Matrix metalloproteinases and the regulation of tissue remodelling. Nat Rev Mol Cell Biol.

[165] Bode W., Fernandez-Catalan C., Tschesche H., Grams F., Nagase H., Maskos K. (1999). Structural properties of matrix metalloproteinases. Cell Mol Life SciCMLS.

[166] Nagase H., Visse R., Murphy G. (2006). Structure and function of matrix metalloproteinases and TIMPs. Cardiovasc Res.

[167] Tallant C., Marrero A., Gomis-Rüth F.X. (2010). Matrix metalloproteinases: fold and function of their catalytic domains. Biochim Biophys Acta (BBA) - Mol Cell Res.

[168] Maskos K., Bode W. (2003). Structural basis of matrix metalloproteinases and tissue inhibitors of metalloproteinases. Mol Biotechnol.

[169] Van Wart H.E., Birkedal-Hansen H. (1990). The cysteine switch: a principle of regulation of metalloproteinase activity with potential applicability to the entire matrix metalloproteinase gene family. Proc Natl Acad Sci USA.

[170] Overall C.M. (2002). Molecular determinants of metalloproteinase substrate specificity. Mol Biotechnol.

[171] Nagase H., Clendeninn N.J., Appelt K. (2001). Matrix metalloproteinase inhibitors in cancer therapy.

[172] Rangasamy G., Ortín C., Zapico R., de Pascual-Teresa (2019). Molecular imaging probes based on matrix metalloproteinase inhibitors (MMPIs). Molecules.

[173] Hu Q., Ecker M. (2021). Overview of MMP-13 as a promising target for the treatment of osteoarthritis. IJMS.

[174] Mannello F., Medda V. (2012). Nuclear localization of Matrix metalloproteinases. Prog Histochem Cytochem.

[175] Mountain D.J.H., Singh M., Menon B., Singh K. (2007). Interleukin-1β increases expression and activity of matrix metalloproteinase-2 in cardiac microvascular endothelial cells: role of PKCα/β1 and MAPKs. Am J Physiol-Cell Physiol.

[176] Henriet P., Emonard H. (2019). Matrix metalloproteinase-2: not (just) a “hero” of the past. Biochimie.

[177] Vincenti M.P., Brinckerhoff C.E. (2007). Signal transduction and cell-type specific regulation of matrix metalloproteinase gene expression: can MMPs be good for you?. J Cell Physiol.

[178] Kobayashi T., Kim H., Liu X., Sugiura H., Kohyama T., Fang Q., Wen F.-Q., Abe S., Wang X., Atkinson J.J., Shipley J.M., Senior R.M., Rennard S.I. (2014). Matrix metalloproteinase-9 activates TGF-β and stimulates fibroblast contraction of collagen gels. Am J Physiol-Lung Cell Mol Physiol.

[179] Pepper M.S. (2001). Role of the matrix metalloproteinase and plasminogen activator–plasmin systems in angiogenesis, arteriosclerosis, thrombosis. Vasc Biol.

[180] Ardi V.C., Van den Steen P.E., Opdenakker G., Schweighofer B., Deryugina E.I., Quigley J.P. (2009). Neutrophil MMP-9 proenzyme, unencumbered by TIMP-1, undergoes efficient activation in vivo and catalytically induces angiogenesis via a basic fibroblast growth factor (FGF-2)/FGFR-2 pathway. J Biol Chem.

[181] Rundhaug J.E. (2005). Matrix metalloproteinases and angiogenesis. J Cell Mol Med.

[182] Bigg H.F., Rowan A.D., Barker M.D., Cawston T.E. (2007). Activity of matrix metalloproteinase-9 against native collagen types I and III. FEBS J.

[183] Barillari G. (2020). The Impact of Matrix Metalloproteinase-9 on the Sequential Steps of the Metastatic Process. IJMS.

[184] Van Doren S.R. (2015). Matrix metalloproteinase interactions with collagen and elastin. Matrix Biol.

[185] Zhao X., Benveniste E.N. (2008). Transcriptional activation of human matrix metalloproteinase-9 gene expression by multiple co-activators. J Mol Biol.

[186] Benbow U., Brinckerhoff C.E. (1997). The AP-1 site and MMP gene regulation: what is all the fuss about?. Matrix Biol.

[187] Sato H., Takino T. (2010). Coordinate action of membrane-type matrix metalloproteinase-1 (MT1-MMP) and MMP-2 enhances pericellular proteolysis and invasion. Cancer Sci.

[188] Mazzieri R., Masiero L., Zanetta L., Monea S., Onisto M., Garbisa S., Mignatti P. (1997). Control of type IV collagenase activity by components of the urokinase–plasmin system: a regulatory mechanism with cell-bound reactants. EMBO J.

[189] H. Nagase, Chapter 158 - matrix metalloproteinase 3/Stromelysin 1, in: N.D. Rawlings, G. Salvesen (Ed.), Handbook of proteolytic enzymes (Third Edition), Academic Press, 2013: pp. 763–74. 10.1016/B978-0-12-382219-2.00158-7.

[190] Okada Y., Nagase H., Harris E.D. (1986). A metalloproteinase from human rheumatoid synovial fibroblasts that digests connective tissue matrix components. Purification and characterization. J Biol Chem.

[191] Woessner J. (1998). The metalloproteinase family. Matrix Metalloproteinases.

[192] Bernardo M.M., Fridman R. (2003). TIMP-2 (tissue inhibitor of metalloproteinase-2) regulates MMP-2 (matrix metalloproteinase-2) activity in the extracellular environment after pro-MMP-2 activation by MT1 (membrane type 1)-MMP. Biochem J.

[193] Gifford V., Itoh Y. (2019). MT1-MMP-dependent cell migration: proteolytic and non-proteolytic mechanisms. Biochem Soc Trans.

[194] Cheng C.-Y., Kuo C.-T., Lin C.-C., Hsieh H.-L., Yang C.-M. (2010). IL-1β induces expression of matrix metalloproteinase-9 and cell migration via a c-Src-dependent, growth factor receptor transactivation in A549 cells. Br J Pharmacol.

[195] Docherty A., Murphy G. (1990). The tissue metalloproteinase family and the inhibitor TIMP: a study using cDNAs and recombinant proteins. Ann Rheum Dis.

[196] Kim E.-M., Hwang O. (2011). Role of matrix metalloproteinase-3 in neurodegeneration. J Neurochem.

[197] Piskór B.M., Przylipiak A., Dąbrowska E., Niczyporuk M., Ławicki S. (2020). Matrilysins and stromelysins in pathogenesis and diagnostics of cancers. CMAR.

[198] Gaire M., Magbanua Z., McDonnell S., McNeil L., Lovett D.H., Matrisian L.M. (1994). Structure and expression of the human gene for the matrix metalloproteinase matrilysin. J Biol Chem.

[199] Shlopov B.V., Lie W.-R., Mainardi C.L., Cole A.A., Chubinskaya S., Hasty K.A. (1997). Osteoarthritic lesions. involvement of three different collagenases. Arthrit Rheumat.

[200] Yan C., Boyd D.D. (2007). Regulation of matrix metalloproteinase gene expression. J Cell Physiol.

[201] Ii M., Yamamoto H., Adachi Y., Maruyama Y., Shinomura Y. (2006). Role of matrix metalloproteinase-7 (Matrilysin) in human cancer invasion, apoptosis, growth, and angiogenesis. Exp Biol Med (Maywood).

[202] Gill S.E., Nadler S.T., Li Q., Frevert C.W., Park P.W., Chen P., Parks W.C. (2016). Shedding of syndecan-1/CXCL1 complexes by matrix metalloproteinase 7 functions as an epithelial checkpoint of neutrophil activation. Am J Respir Cell Mol Biol.

[203] Park H.I., Ni J., Gerkema F.E., Liu D., Belozerov V.E., Sang Q.-X.A. (2000). Identification and characterization of human endometase (matrix metalloproteinase-26) from endometrial tumor. J Biol Chem.

[204] Marchenko G.N., Marchenko N.D., Leng J., Strongin A.Y. (2002). Promoter characterization of the novel human matrix metalloproteinase-26 gene: regulation by the T-cell factor-4 implies specific expression of the gene in cancer cells of epithelial origin. Biochem J.

[205] Barbolina M.V., Stack M.S. (2008). Membrane type 1-matrix metalloproteinase: substrate diversity in pericellular proteolysis. Semin Cell Dev Biol.

[206] Ohuchi E., Imai K., Fujii Y., Sato H., Seiki M., Okada Y. (1997). Membrane Type 1 matrix metalloproteinase digests interstitial collagens and other extracellular matrix macromolecules. J Biol Chem.

[207] Knäuper V., Bailey L., Worley J.R., Soloway P., Patterson M.L., Murphy G. (2002). Cellular activation of proMMP-13 by MT1-MMP depends on the C-terminal domain of MMP-13. FEBS Lett.

[208] Szabova L., Yamada S.S., Wimer H., Chrysovergis K., Ingvarsen S., Behrendt N., Engelholm L.H., Holmbeck K. (2009). MT1-MMP and Type II collagen specify skeletal stem cells and their bone and cartilage progeny. J Bone Min Res.

[209] Zhu L., Tang Y., Li X.-Y., Keller E.T., Yang J., Cho J.-S., Feinberg T.Y., Weiss S.J. (2020). Osteoclast-mediated bone resorption is controlled by a compensatory network of secreted and membrane-tethered metalloproteinases. Sci Transl Med.

[210] Ando A., Hagiwara Y., Tsuchiya M., Onoda Y., Suda H., Chimoto E., Itoi E. (2009). Increased expression of metalloproteinase-8 and-13 on articular cartilage in a rat immobilized knee model. Tohoku J Exp Med.

[211] Mitsui H., Tsuchiya N., Okinaga S., Matsuta K., Yoshimura K., Nishimura A. (2001). Expression of membrane-type matrix metalloproteinases in synovial tissue from patients with rheumatoid arthritis or osteoarthritis. Mod Rheumatol.

[212] Pardo A., Selman M. (2005). MMP-1: the elder of the family. Int J Biochem Cell Biol.

[213] Itoh Y. (2015). Membrane-type matrix metalloproteinases: their functions and regulations. Matrix Biol.

[214] Itoh Y., Seiki M. (2006). MT1-MMP: a potent modifier of pericellular microenvironment. J Cell Physiol.

[215] Emonard H., Bellon G., Troeberg L., Berton A., Robinet A., Henriet P., Marbaix E., Kirkegaard K., Patthy L., Eeckhout Y., Nagase H., Hornebeck W., Courtoy P.J. (2004). Low density lipoprotein receptor-related protein mediates endocytic clearance of pro-MMP-2·TIMP-2 complex through a thrombospondin-independent mechanism*. J Biol Chem.

[216] Sottrup-Jensen L. (1989). α-Macroglobulins: structure, shape, and mechanism of proteinase complex formation. J Biol Chem.

[217] Strickland D.K., Ashcom J.D., Williams S., Burgess W.H., Migliorini M., Argraves W.S. (1990). Sequence identity between the alpha 2-macroglobulin receptor and low density lipoprotein receptor-related protein suggests that this molecule is a multifunctional receptor. J Biol Chem.

[218] Arpino V., Brock M., Gill S.E. (2015). The role of TIMPs in regulation of extracellular matrix proteolysis. Matrix Biol.

[219] Sulkala M., Tervahartiala T., Sorsa T., Larmas M., Salo T., Tjäderhane L. (2007). Matrix metalloproteinase-8 (MMP-8) is the major collagenase in human dentin. Arch Oral Biol.

[220] Quillard T., Araújo H.A., Franck G., Tesmenitsky Y., Libby P. (2014). MMP-13 predominates over MMP-8 as the functional interstitial collagenase in mouse atheromata. Arterioscler Thromb Vasc Biol.

[221] Cox J.H., Starr A.E., Kappelhoff R., Yan R., Roberts C.R., Overall C.M. (2010). Matrix metalloproteinase 8 deficiency in mice exacerbates inflammatory arthritis through delayed neutrophil apoptosis and reduced caspase 11 expression. Arthrit Rheumat.

[222] Murphy G. (2011). Tissue inhibitors of metalloproteinases. Genome Biol.

[223] Mauviel A. (1993). Cytokine regulation of metalloproteinase gene expression. J Cell Biochem.

[224] Lenglet S., Mach F., Montecucco F. (2013). Role of matrix metalloproteinase-8 in atherosclerosis. Mediat Inflamm.

[225] Owen C.A., Hu Z., Lopez-Otin C., Shapiro S.D. (2004). Membrane-bound matrix metalloproteinase-8 on activated polymorphonuclear cells is a potent, tissue inhibitor of metalloproteinase-resistant collagenase and serpinase1. J Immunol.

[226] Pirilä E., Korpi J.T., Korkiamäki T., Jahkola T., Gutierrez-Fernandez A., Lopez-Otin C., Saarialho-Kere U., Salo T., Sorsa T. (2007). Collagenase-2 (MMP-8) and matrilysin-2 (MMP-26) expression in human wounds of different etiologies. Wound Repair Regen.

[227] Moldovan F., Pelletier J.-P., Hambor J., Cloutier J.-M., Martel-Pelletier J. (1997). Collagenase-3 (matrix metalloprotease 13) is preferentially localized in the deep layer of human arthritic cartilage in situ. In vitro mimicking effect by transforming growth factor β. Arthrit Rheumat.

[228] G.A. Cabral-Pacheco, I. Garza-Veloz, C. Castruita-De la Rosa, J.M. Ramirez-Acuña, B.A. Perez-Romero, J.F. Guerrero-Rodriguez, N. Martinez-Avila, M.L. Martinez-Fierro, The roles of matrix metalloproteinases and their inhibitors in human diseases, IJMS. 21 (2020) 9739. 10.3390/ijms21249739. PMC7767220 33419373

[229] Mengshol J.A., Vincenti M.P., Coon C.I., Barchowsky A., Brinckerhoff C.E. (2000). Interleukin-1 induction of collagenase 3 (matrix metalloproteinase 13) gene expression in chondrocytes requires p38, c-jun N-terminal kinase, and nuclear factor κB: differential regulation of collagenase 1 and collagenase 3. Arthrit Rheumat.

[230] Vincenti M.P., Brinckerhoff C.E. (2002). Transcriptional regulation of collagenase (MMP-1, MMP-13) genes in arthritis: integration of complex signaling pathways for the recruitment of gene-specific transcription factors. Arthritis Res Ther.

[231] Chung L., Dinakarpandian D., Yoshida N., Lauer-Fields J.L., Fields G.B., Visse R., Nagase H. (2004). Collagenase unwinds triple-helical collagen prior to peptide bond hydrolysis. EMBO J.

[232] Knäuper V., Cowell S., Smith B., López-Otin C., O'Shea M., Morris H., Zardi L., Murphy G. (1997). The role of the C-terminal domain of human collagenase-3 (MMP-13) in the activation of procollagenase-3, substrate specificity, and tissue inhibitor of metalloproteinase interaction. J Biol Chem.

[233] Heathfield T.F., Önnerfjord P., Dahlberg L., Heinegård D. (2004). Cleavage of fibromodulin in cartilage explants involves removal of the N-terminal tyrosine sulfate-rich region by proteolysis at a site that is sensitive to matrix metalloproteinase-13. J Biol Chem.

[234] Inada M., Wang Y., Byrne M.H., Rahman M.U., Miyaura C., López-Otín C., Krane S.M. (2004). Critical roles for collagenase-3 (Mmp13) in development of growth plate cartilage and in endochondral ossification. Proc Natl Acad Sci USA.

[235] Mitchell P.G., Magna H.A., Reeves L.M., Lopresti-Morrow L.L., Yocum S.A., Rosner P.J., Geoghegan K.F., Hambor J.E. (1996). Cloning, expression, and type II collagenolytic activity of matrix metalloproteinase-13 from human osteoarthritic cartilage. J Clin Investig.

[236] Reboul P., Pelletier J.P., Tardif G., Cloutier J.M., Martel-Pelletier J. (1996). The new collagenase, collagenase-3, is expressed and synthesized by human chondrocytes but not by synoviocytes. A role in osteoarthritis. J Clin Investig.

[237] Alameddine H.S., Morgan J.E. (2016). Matrix metalloproteinases and tissue inhibitor of metalloproteinases in inflammation and fibrosis of skeletal muscles. JND.

[238] Shipley J.M., Doyle G.A.R., Fliszar C.J., Ye Q.-Z., Johnson L.L., Shapiro S.D., Welgus H.G., Senior R.M. (1996). The structural basis for the elastolytic activity of the 92-kDa and 72-kDa gelatinases. J Biol Chem.

[239] Steffensen B., Wallon U.M., Overall C.M. (1995). Extracellular matrix binding properties of recombinant fibronectin type II-like modules of human 72-kDa gelatinase/Type IV collagenase. J Biol Chem.

[240] Grzela T., Krejner A., Litwiniuk M. (2016). Matrix metalloproteinases in the wound microenvironment: therapeutic perspectives. CWCMR.

[241] Morgunova E., Tuuttila A., Bergmann U., Isupov M., Lindqvist Y., Schneider G., Tryggvason K. (1999). Structure of human pro-matrix metalloproteinase-2: activation mechanism revealed. Science (1979).

[242] Knäuper V., Will H., López-Otin C., Smith B., Atkinson S.J., Stanton H., Hembry R.M., Murphy G. (1996). Cellular mechanisms for human procollagenase-3 (MMP-13) activation. J Biol Chem.

[243] Yamamoto K., Okano H., Miyagawa W., Visse R., Shitomi Y., Santamaria S., Dudhia J., Troeberg L., Strickland D.K., Hirohata S., Nagase H. (2016). MMP-13 is constitutively produced in human chondrocytes and co-endocytosed with ADAMTS-5 and TIMP-3 by the endocytic receptor LRP1. Matrix Biol.

[244] Milaras C., Lepetsos P., Dafou D., Potoupnis M., Tsiridis E. (2021). Association of matrix metalloproteinase (MMP) gene polymorphisms with knee osteoarthritis: a review of the literature. Cureus.

[245] Clark I.M., Cawston T.E. (1989). Fragments of human fibroblast collagenase. Purification and characterization. Biochem J.

[246] Birkedal-Hansen H., Moore W.G.I., Bodden M.K., Windsor L.J., Birkedal-Hansen B., DeCarlo A., Engler J.A. (1993). Matrix metalloproteinases: a review. Crit Rev Oral Biol Med.

[247] Allan J.A., Docherty A.J.P., Barker P.J., Huskisson N.S., Reynolds J.J., Murphy G. (1995). Binding of gelatinases A and B to type-I collagen and other matrix components. Biochem J.

[248] Vandooren J., Van den Steen P.E., Opdenakker G. (2013). Biochemistry and molecular biology of gelatinase B or matrix metalloproteinase-9 (MMP-9): the next decade. Crit Rev Biochem Mol Biol.

[249] Viappiani S., Nicolescu A.C., Holt A., Sawicki G., Crawford B.D., León H., van Mulligen T., Schulz R. (2009). Activation and modulation of 72kDa matrix metalloproteinase-2 by peroxynitrite and glutathione. Biochem Pharmacol.

[250] Itoh Y., Takamura A., Ito N., Maru Y., Sato H., Suenaga N., Aoki T., Seiki M. (2001). Homophilic complex formation of MT1-MMP facilitates proMMP-2 activation on the cell surface and promotes tumor cell invasion. EMBO J.

[251] Patterson M.L., Atkinson S.J., Knäuper V., Murphy G. (2001). Specific collagenolysis by gelatinase A, MMP-2, is determined by the hemopexin domain and not the fibronectin-like domain. FEBS Lett.

[252] Billinghurst R.C., Dahlberg L., Ionescu M., Reiner A., Bourne R., Rorabeck C., Mitchell P., Hambor J., Diekmann O., Tschesche H. (1997). Enhanced cleavage of type II collagen by collagenases in osteoarthritic articular cartilage. J Clin Investig.

[253] DeCoux A., Lindsey M.L., Villarreal F., Garcia R.A., Schulz R. (2014). Myocardial matrix metalloproteinase-2: inside out and upside down. J Mol Cell Cardiol.

[254] Amălinei C., Căruntu I.-D., Bălan R.A. (2007). Biology of metalloproteinases. Rom J Morphol Embryol.

[255] Ramos-DeSimone N., Hahn-Dantona E., Sipley J., Nagase H., French D.L., Quigley J.P. (1999). Activation of matrix metalloproteinase-9 (MMP-9) via a converging plasmin/stromelysin-1 cascade enhances tumor cell invasion. J Biol Chem.

[256] Nikolov A., Popovski N. (2021). Role of gelatinases MMP-2 and MMP-9 in healthy and complicated pregnancy and their future potential as preeclampsia biomarkers. Diagnostics.

[257] Aimes R.T., Quigley J.P. (1995). Matrix metalloproteinase-2 is an interstitial collagenase. J Biol Chem.

[258] Galasso O., Familiari F., De Gori M., Gasparini G. (2012). Recent findings on the role of gelatinases (matrix metalloproteinase-2 and -9) in osteoarthritis. Adv Orthop.

[259] Van Lint P., Libert C. (2006). Matrix metalloproteinase-8: cleavage can be decisive. Cytokine Growth Factor Rev.

[260] Yabluchanskiy A., Ma Y., Iyer R.P., Hall M.E., Lindsey M.L. (2013). Matrix metalloproteinase-9: many shades of function in cardiovascular disease. Physiology.

[261] Huang H. (2018). Matrix metalloproteinase-9 (MMP-9) as a cancer biomarker and MMP-9 biosensors: recent advances. Sensors.

[262] Ayuk S.M., Abrahamse H., Houreld N.N. (2016). The role of matrix metalloproteinases in diabetic wound healing in relation to photobiomodulation. J Diabetes Res.

[263] Van den Steen P.E., Dubois B., Nelissen I., Rudd P.M., Dwek R.A., Opdenakker G. (2002). Biochemistry and molecular biology of gelatinase B or matrix metalloproteinase-9 (MMP-9). Crit Rev Biochem Mol Biol.

[264] Dolińska E., Pietruska M., Dymicka-Piekarska V., Milewski R., Sculean A. (2022). Matrix metalloproteinase 9 (MMP-9) and interleukin-8 (IL-8) in gingival crevicular fluid after regenerative therapy in periodontal intrabony defects with and without systemic antibiotics—randomized clinical trial. Pathogens.

[265] Mixon A., Savage A., Bahar-Moni A.S., Adouni M., Faisal T. (2021). An in vitro investigation to understand the synergistic role of MMPs-1 and 9 on articular cartilage biomechanical properties. Sci Rep.

[266] Farina A., Mackay A. (2014). Gelatinase B/MMP-9 in tumour pathogenesis and progression. Cancers (Basel).

[267] Iyer S., Visse R., Nagase H., Acharya K.R. (2006). Crystal structure of an active form of human MMP-1. J Mol Biol.

[268] Van den Steen P.E., Wuyts A., Husson S.J., Proost P., Van Damme J., Opdenakker G. (2003). Gelatinase B/MMP-9 and neutrophil collagenase/MMP-8 process the chemokines human GCP-2/CXCL6, ENA-78/CXCL5 and mouse GCP-2/LIX and modulate their physiological activities. Eur J Biochem.

[269] Dunsmore S.E., Saarialho-Kere U.K., Roby J.D., Wilson C.L., Matrisian L.M., Welgus H.G., Parks W.C. (1998). Matrilysin expression and function in airway epithelium. J Clin Invest.

[270] Wilson C.L., Matrisian L.M. (1996). Matrilysin: an epithelial matrix metalloproteinase with potentially novel functions. Int J Biochem Cell Biol.

[271] Mengshol J.A., Mix K.S., Brinckerhoff C.E. (2002). Matrix metalloproteinases as therapeutic targets in arthritic diseases: bull's-eye or missing the mark?. Arthr Rheumat.

[272] Murphy G., Allan J.A., Willenbrock F., Cockett M.I., O'Connell J.P., Docherty A.J. (1992). The role of the C-terminal domain in collagenase and stromelysin specificity. J Biol Chem.

[273] Haro H., Crawford H.C., Fingleton B., Shinomiya K., Spengler D.M., Matrisian L.M. (2000). Matrix metalloproteinase-7–dependent release of tumor necrosis factor-α in a model of herniated disc resorption. J Clin Invest.

[274] Meyer B.S., Rademann J. (2012). Extra- and intracellular imaging of human matrix metalloprotease 11 (hMMP-11) with a cell-penetrating FRET substrate. J Biol Chem.

[275] Gimeno A., Beltrán-Debón R., Mulero M., Pujadas G., Garcia-Vallvé S. (2020). Understanding the variability of the S1′ pocket to improve matrix metalloproteinase inhibitor selectivity profiles. Drug Discov Today.

[276] Saarialho-Kere U.K., Crouch E.C., Parks W.C. (1995). Matrix metalloproteinase matrilysin is constitutively expressed in adult human exocrine epithelium. J Investig Dermatol.

[277] Suárez H., López-Martín S., Toribio V., Zamai M., Hernández-Riquer M.V., Genís L., Arroyo A.G., Yáñez-Mó M. (2020). Regulation of MT1-MMP activity through its association with ERMs. Cells.

[278] Knox J.D., Boreham D.R., Walker J.-A., Morrison D.P., Matrisian L.M., Nagle R.B., Bowden G.T. (1996). Mapping of the metalloproteinase gene matrilysin (MMP7) to human chromosome 11q21→q22. Cytogenet Genome Res.

[279] SEIKI M. (1999). Membrane-type matrix metalloproteinases. APMIS.

[280] English W.R., Holtz B., Vogt G., Knäuper V., Murphy G. (2001). Characterization of the role of the “MT-loop. J Biol Chem.

[281] Ra H.-J., Parks W.C. (2007). Control of matrix metalloproteinase catalytic activity. Matrix Biol.

[282] Devarajan P., Mookhtiar K., Van Wart H., Berliner N. (1991). Structure and expression of the cDNA encoding human neutrophil collagenase. Blood.

[283] Brew K., Dinakarpandian D., Nagase H. (2000). Tissue inhibitors of metalloproteinases: evolution, structure and function11Dedicated to Professor H. Neurath on the occasion of his 90th birthday. Biochim Biophys Acta (BBA) - Prot Struct Mol Enzymol.

[284] Costanzo L., Soto B., Meier R., Geraghty P. (2022). The biology and function of tissue inhibitor of metalloproteinase 2 in the lungs. Pulm Med.

[285] Brew K., Nagase H. (2010). The tissue inhibitors of metalloproteinases (TIMPs): an ancient family with structural and functional diversity. Biochim Biophys Acta (BBA) - Mol Cell Res.

[286] Justo B.L., Jasiulionis M.G. (2021). Characteristics of TIMP1, CD63, and β1-integrin and the functional impact of their interaction in cancer. IJMS.

[287] Toricelli M., Melo F., Hunger A., Zanatta D., Strauss B., Jasiulionis M. (2017). Timp1 promotes cell survival by activating the PDK1 signaling pathway in melanoma. Cancers (Basel).

[288] Wang T., Yamashita K., Iwata K., Hayakawa T. (2002). Both tissue inhibitors of metalloproteinases-1 (TIMP-1) and TIMP-2 activate Ras but through different pathways. Biochem Biophys Res Commun.

[289] Stetler-Stevenson W.G. (2008). Tissue inhibitors of metalloproteinases in cell signaling: metalloproteinase-independent biological activities. Sci Signal.

[290] Furman D., Campisi J., Verdin E., Carrera-Bastos P., Targ S., Franceschi C., Ferrucci L., Gilroy D.W., Fasano A., Miller G.W., Miller A.H., Mantovani A., Weyand C.M., Barzilai N., Goronzy J.J., Rando T.A., Effros R.B., Lucia A., Kleinstreuer N., Slavich G.M. (2019). Chronic inflammation in the etiology of disease across the life span. Nat Med.

[291] Sagi I., Talmi-Frank D., Arkadash V., Papo N., Mohan V. (2016). Matrix metalloproteinase protein inhibitors: highlighting a new beginning for metalloproteinases in medicine. MNM.

[292] Nagase H., Brew K. (2002). Engineering of tissue inhibitor of metalloproteinases mutants as potential therapeutics. Arthritis Res Ther.

[293] Cerofolini L., Fragai M., Luchinat C. (2019). Mechanism and inhibition of matrix metalloproteinases. CMC.

[294] Gomis-Rüth F.X. (2003). Structural aspects of the metzincin clan of metalloendopeptidases. MB.

[295] Clark I.M., Swingler T.E., Sampieri C.L., Edwards D.R. (2008). The regulation of matrix metalloproteinases and their inhibitors. Int J Biochem Cell Biol.

[296] Fan D., Kassiri Z. (2020). Biology of tissue inhibitor of metalloproteinase 3 (TIMP3), and its therapeutic implications in cardiovascular pathology. Front Physiol.

[297] Levin M., Udi Y., Solomonov I., Sagi I. (2017). Next generation matrix metalloproteinase inhibitors — Novel strategies bring new prospects. Biochim Biophys Acta (BBA) - Mol Cell Res.

[298] Vincenti M.P., Clark I.M. (2001). Matrix metalloproteinase protocols.

[299] Troeberg L., Fushimi K., Scilabra S.D., Nakamura H., Dive V., Thøgersen I.B., Enghild J.J., Nagase H. (2009). The C-terminal domains of ADAMTS-4 and ADAMTS-5 promote association with N-TIMP-3. Matrix Biol.

[300] Kashiwagi M., Tortorella M., Nagase H., Brew K. (2001). TIMP-3 is a potent inhibitor of aggrecanase 1 (ADAM-TS4) and aggrecanase 2 (ADAM-TS5). J Biol Chem.

[301] Grässel S., Muschter D. (2020). Recent advances in the treatment of osteoarthritis. F1000Res.

